# Intraductal photothermal ablation: a noninvasive approach for early breast cancer treatment and prevention

**DOI:** 10.7150/thno.97968

**Published:** 2024-07-01

**Authors:** Jianhua Liu, Biao Huang, Yan Rao, Liantao Guo, Cheguo Cai, Dongcheng Gao, Deguang Kong, Guannan Wang, Yao Xiong, Ran Cui, Mingxi Zhang, Chuang Chen

**Affiliations:** 1 Department of Breast and Thyroid Surgery, Renmin Hospital of Wuhan University, Wuhan, Hubei 430060, PR China.; 2 Department of Breast Surgery, Hubei Cancer Hospital, Tongji Medical College, Huazhong University of Science and Technology, Hubei Provincial Clinical Research Center for Breast Cancer, Wuhan Clinical Research Center for Breast Cancer. No.116 Zhuo Daoquan South Road, Wuhan, Hubei 430079, PR China.; 3 College of Chemistry and Molecular Sciences, Wuhan University, 430072 Wuhan, PR China.; 4 Animal Biosafety Level III Laboratory at the Center for Animal Experiment, Wuhan University School of Medicine, Wuhan, 430071, PR China.; 5 Department of Thyroid and Breast Surgery, Frontier Science Center for Immunology and Metabolism, Medical Research Institute, Zhongnan Hospital of Wuhan University, Wuhan University, Wuhan, 430071, PR China.; 6 Lombardi Comprehensive Cancer Center, Georgetown University, 3970 Reservoir Rd NW, New Research Building, Room E204, Washington, D.C. 20007, USA.; 7 State Key Laboratory of Advanced Technology for Materials Synthesis and Processing, Wuhan University of Technology, 430070 Wuhan, PR China.

**Keywords:** Breast carcinoma, Nanoparticles, Photothermal ablation, Intraductal therapy, Non-invasive surgery, Mammary gland, Immunoenhancement

## Abstract

**Background:** Innovative treatment strategies for early-stage breast cancer (BC) are urgently needed. Tumors originating from mammary ductal cells present an opportunity for targeted intervention.

**Methods:** We explored intraductal therapy via natural nipple openings as a promising non-invasive approach for early BC. Using functional Near-infrared II (NIR-II) nanomaterials, specifically NIR-IIb quantum dots conjugated with Epep polypeptide for ductal cell targeting, we conducted *in situ* imaging and photothermal ablation of mammary ducts. Intraductal administration was followed by stimulation with an 808 nm laser.

**Results:** This method achieved precise ductal destruction and heightened immunological responses in the microenvironment. The technique was validated in mouse models of triple-negative BC and a rat model of ductal carcinoma *in situ*, demonstrating promising therapeutic potential for localized BC treatment and prevention.

**Conclusion:** Our study demonstrated the effectiveness of NIR-II nanoprobes in guiding non-invasive photothermal ablation of mammary ducts, offering a compelling avenue for early-stage BC therapy.

## Introduction

Breast cancer (BC) is one of the most prevalent malignant tumors globally 1. Over recent decades, BC prognosis has improved considerably through early screening and effective systemic therapies. Surgical approaches have evolved from wide resections to minimally invasive paradigms 2. Presently, lumpectomy coupled with radiotherapy remains the gold standard for local BC treatment. However, due to the variability in the number of visible and occult lesions, many patients with early-stage BC, such as ductal carcinoma *in situ* (DCIS) or micro-invasion, undergo mastectomy. Additionally, some patients, even after a pathologic complete response (pCR), may require extensive resections, and high-risk individuals may opt for prophylactic mastectomy 3-6. Recent preclinical and clinical studies have explored de-escalation or omission of breast surgery for these patients 7-10. An urgent need exists for efficient and precise non-invasive strategies in BC personalized local therapy.

Most breast tumors originate from ductal epithelial cells of the mammary gland 7,11-14. Targeting these cells and their microenvironment is imperative for BC local therapy. Intraductal (i.duc) therapy has emerged as a promising non-invasive approach 15. Therapeutic agents are introduced directly into mammary ducts *via* the natural openings of the nipple, offering an effective and minimally invasive BC therapy approach. Studies, including our own, have assessed the advantages of i.duc therapy over traditional systemic administration of chemotherapeutics, targeted agents, endocrine drugs, and nanomedicines 16-18.

In recent decades, thermal ablation has been a leading strategy for local cancer therapy, enhancing immunological effects 19-23. Yet, suboptimal precision and efficiency persist due to limited thermal imaging resolution 22-27. Quantum dots (QDs) emitting in the near-infrared-IIb (NIR-IIb, 1500-1700 nm) range offer the potential for precision medicine 24,26,27. Our prior studies indicated that NIR-II QD-based nanoprobes are potent tools for non-invasive deep tissue imaging and *in vivo* immune cell monitoring 28. Recently, we showed that NIR-II QD-based nanoprobes enhance immunogenic responses in radiotherapy 29. Hence, we hypothesized that NIR-IIb QDs can be utilized for targeted photothermal ablation of mammary ducts *via* the i.duc route, providing a non-invasive approach for imaging-guided BC therapy.

**Figure 1:**
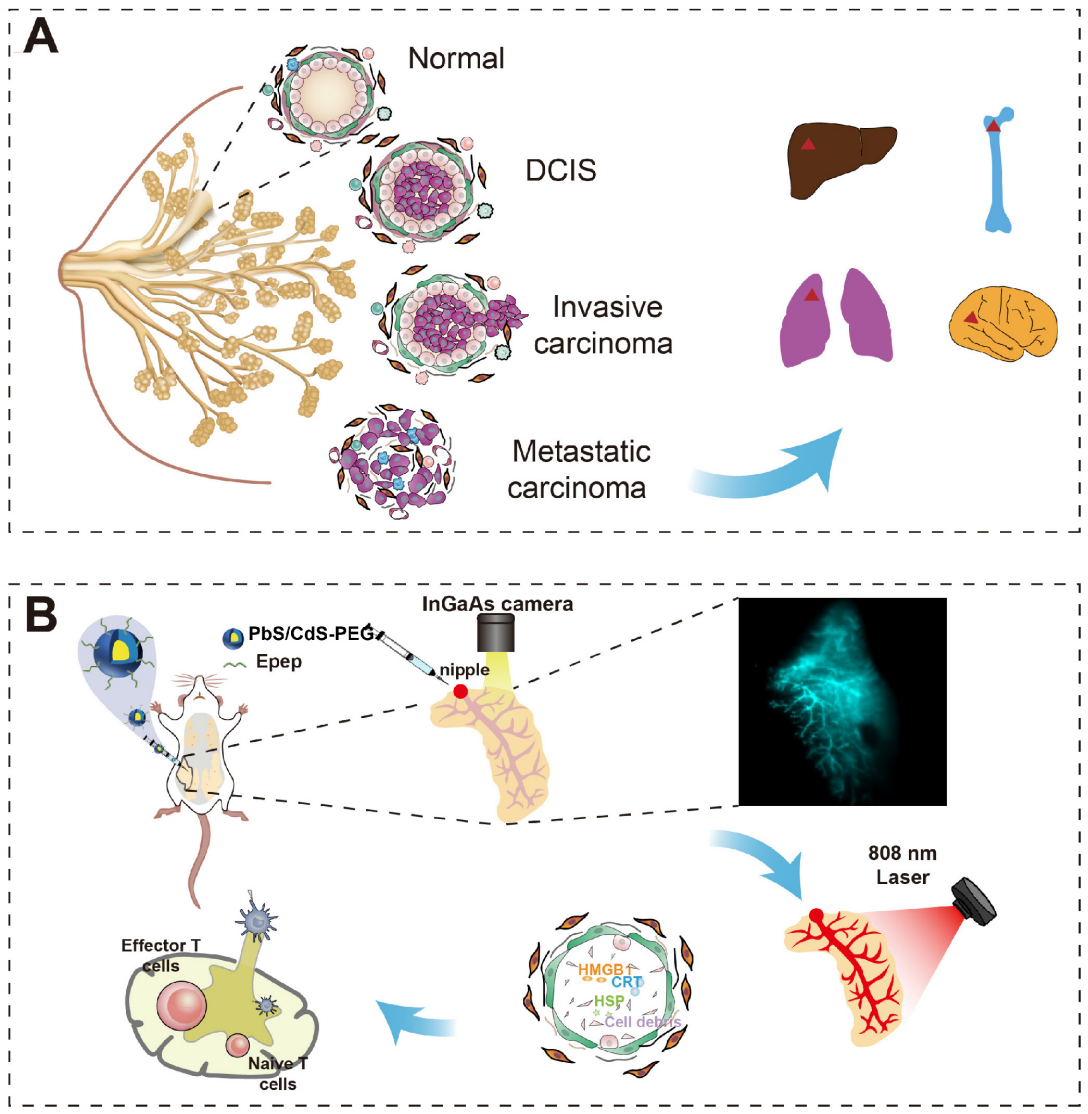
** NIR-IIb imaging-guided photothermal ablation of mammary ducts *in situ* for noninvasive BC therapy. A** Breast tumor arise from clonal growth of ductal epithelial cells within mammary ducts and can progress to invasion and metastasis in the presence of the tumor microenvironment. Therefore, precise intervention at the mammary ducts, the origin of tumor cells, offers a potential non-invasive approach for BC therapy. **B** Intraductal administration of NIR-IIb PbS/CdS-PEG QDs coupled with Epep polypeptide (targeting mammary duct epithelial cells) enables high-resolution thermal imaging of the entire mammary ductal tree. This approach ensures exceptional accuracy and efficiency in thermally ablating the mammary ducts. Furthermore, targeted ductal ablation holds promise for eliciting antitumor effects with enhanced immune responses.

This study established the efficacy of NIR-IIb QDs for targeted mammary duct imaging and photothermal ablation *via* i.duc therapy (Figure 1). The selection of Epep polypeptide for targeting mammary duct epithelial cells and the preparation of PbS/CdS-PEG QDs conjugated with Epep polypeptide (PbS/CdS-PEG-Epep) enabled *in situ* imaging and visible ablation of mammary ducts under laser irradiation. The precise and efficient ablation elicited activated immune responses. In animal models of triple-negative BC (TNBC) and DCIS, the destruction of mammary ducts, combined with enhanced immunological effects, resulted in remarkable anti-tumor outcomes for both BC treatment and prevention. We conclude that NIR-IIb nanoprobes for imaging-guided photothermal ablation of mammary ducts *via* the i.duc route offer an accurate and efficient non-invasive paradigm for BC therapy.

## Materials and Methods

### Chemicals

Lead chloride (PbCl_2_, 99%, powder) and cadmium oxide (CdO, 99.99%, power) were purchased from Alfa. Sulfur (S, 99.999%, powder) was purchased from Adamas. Oleylamine (OLA, technical grade, 70%), oleic acid (OA, technical grade, 90%), 1-octadecene (ODE), 2-(N-morpholino) ethanesulfonic acid (MES) hydrate, poly (acrylic acid) (Mw~1,800), N, Nʹ-dicyclohexylcarbodiimide (DCC), N-(3-dimethylaminopropyl)-Nʹ-ethylcarbodiimide hydrochloride (EDC), and Epep peptides were synthesized to order by ChinaPeptides, Inc (Shanghai, China) using Fmoc chemistry. Peptides were provided at > 95 % purity, and purity and structure were confirmed with RP-HPLC and ESI-MS. Phosphate-buffered saline (PBS) was purchased from Hyclone. 8-Arm PEG-amine (MW ~40 K) was purchased from Advanced BioChemicals. Other reagents were of analytical grade.

### Synthesis of PbS/CdS-PEG QDs

PbS/CdS-PEG core-shell QDs were synthesized as previously described 28. Sulfur (5 mmol, 0.08 g) and OLA (7.5 mL) were mixed in a two-neck flask at 120 °C for 30 min under 0.1 MPa vacuum to form the sulfur precursor solution. PbCl_2_ (3 mmol, 0.834 g) and OLA (7.5 mL) were mixed in a three-neck flask at 120 °C for 30 min and then heated to 160 °C under 0.1 MPa vacuum to form the lead precursor solution. The sulfur precursor (2.25 mL) was quickly injected into the lead precursor under stirring at 160 °C and maintained for 1 h. Subsequently, the growth reaction was quenched by introducing cold hexane (10 mL) into the reaction solution. The products were separated and purified by adding excess ethanol and OA and collected by centrifugation. After repeating the precipitation procedure with OA 3 times, the PbS QDs were re-suspended in ODE. Next, CdO (9.2 mmol, 1.2 g), OA (8 mL), and ODE (20 mL) were heated to 200 °C under 0.1 MPa vacuum. The mixture was cooled down to 100 °C and stabilized for 30 min, 5 mL of PbS QDs was bubbled with Ar for 5 min, and then injected into the Cd precursor under stirring at 100 °C for 30 min. The growth reaction was terminated by introducing cold hexane (5 mL) into the reaction solution. PbS/CdS-PEG QDs were precipitated with excess ethanol and re-dispersed in hexane.

### Modification of QDs with oleyamine-branched polyacrylic acid (OPA)

The QDs were modified according to our previously described methods 29. Polyacrylic acid (average Mw ~1,800, 0.9 g) and DCC (1.56 g) were mixed into a round-bottom flask, and N, N-dimethylformamide (DMF) (10 mL) was added to dissolve the mixture. Subsequently, OLA (1.2 mL, molar ratio of OLA to PAA was 30%) was added dropwise into the reaction flask. The mixture solution was stirred for ~16h, and 0.5 M HCl (50 mL) was added. The precipitate was isolated by centrifugation and re-dissolved in methanol (3 mL). Then, 1 M HCl (20 mL) was added to the solution and the precipitate was separated by centrifugation. This procedure was repeated at least 5 times. The precipitate was dissolved in chloroform (5 mL) and washed with 1 M HCl (10 mL). The organic phase was collected and dried with anhydrous Na_2_SO_4_. Chloroform was removed under vacuum, and OPA was collected with an average MW of ~3,000 determined by gel permeation chromatography.

For surface modification, PbS/CdS-PEG QDs (5.0 mg) were added to the solution (15 mg OPA dissolved in 2.0 mL chloroform). The mixture was stirred for 30 min at room temperature and the solvent was removed by a rotary evaporator under vacuum. The products were dissolved completely in 2 mL of 50 mM sodium carbonate solution under sonication. The QDs were separated by ultracentrifugation at 16,000*g* for 1 h and dissolved in 2 mL pH 8.5 MES buffer (0.01 M).

### Preparation of nanoprobes used *in vivo*

The 8-Arm PEG-amine (6 mg) and Epep peptides (200 µg) were dissolved in 200 µL pH 8.5 MES buffer (0.01 M) and gradually added to the OPA-modified QDs solution (2.5 mg) with stirring. EDC (3mg) was dissolved in 50 µL pH 8.5 MES buffer, gradually added to the solution with stirring, and kept at room temperature (25 ℃) overnight. The PbS/CdS-PEG-Epep QDs were purified by ultracentrifugation. Finally, the PbS/CdS-PEG-Epep QDs were dissolved in 1× PBS and stored at 4°C.

### Nanoprobe characterization

Transmission electron microscope (TEM) images were collected using a JEM-2100F electron microscope (JEOL) (operating at a 200 kV accelerating voltage). EDS data were obtained by using an EDX spectrometry (EDAX Inc.) equipped with a JEM 2010F microscope. The NIR fluorescence spectra were measured using an FLS1000 fluorescence spectrometer with a Xenon lamp exciter and a PM1700 near infrared photomultiplier detector (Edinburgh Instruments). The zeta potentials and DLS data were recorded on a Malvern Nano-ZS ZEN3600 zetasizer. The spectra were obtained from a Lambda 750 S UV-Vis-NIR spectrometer (Malvern).

### Calculation of photothermal conversion efficiency

Photothermal conversion efficiency was measured and calculated according to our previous study 30**.** Briefly, heating and cooling experiments were carried out to calculate the photothermal conversion efficiency of PbS/CdS-PEG-Epep. A 1 mL aliquot of PbS/CdS-PEG-Epep (1.75 mg mL^-1^) aqueous solution was placed in a 1.5 mL EP tube and irradiated with an 808 nm laser for 10 min at 1.5 W cm^-2^. A temperature sensor was used to monitor the temperature change of the solution in real-time. After irradiation, the laser was turned off, and the dropping temperature was monitored until the initial temperature returned. Repeated heating and cooling experiments (5 cycles) were carried out to study the photothermal stability of PbS/CdS-PEG-Epep. Photothermal conversion efficiency was defined by the following formula:


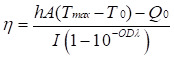

(1)

where ℎ is the heat transfer coefficient, which acts as a proportional constant. *A* denotes the surface area of the container holding the solution. *T*_0_ and *T*_max_​ represent the ambient temperature and the maximum temperature of the solution after laser irradiation, respectively. *Q*_0_ indicates the heat energy produced by the solvent during laser irradiation, calculable by measuring the temperature rise in pure water post-irradiation. *I* signifies the laser power, and *OD_λ_*_​_ refers to the absorbance of the solution at a wavelength of *λ* nm.

### *In vivo* high-resolution NIR-II fluorescence imaging for mammary ducts

Balb/c mice and Sprague Dawley (SD) rats received i.duc injections of 20 μL and 40 μL PbS/CdS-PEG-Epep (1.75 mg mL^-1^), respectively. NIR-IIb images of the mammary ducts of mice and rats were recorded by a NIR-II small animal InGaAs camera (NIR vana, Princeton Instruments). The high-resolution fluorescence imaging of the mammary ducts in mice or rats was performed with a high-magnification lens (10× objective). The excitation power density was 25 or 50 mW cm^-2^ provided by an 808 nm laser. The exposure time was 200 milliseconds (ms).

### Targeting of PbS/CdS-PEG-Epep *in vitro*

As reported previously 30, a 35 mm^2^ culture dish was used to cultivate HC11 cells (mouse ductal breast epithelial cells) for 24 hours. 300 g/mL of PbS/CdS-PEG- Epep or PbS/CdS-PEG was added after discarding the medium, and the mixture was co-incubated for 30 min. Following two rounds of washing with PBS buffer, the cells were imaged on an NIR-II setup with 808 nm excitation and 980 nm long pass + 1500 nm long pass emission filters for NIR-IIb fluorescent channels.

### Animal experiments

Mice (8 - 10 weeks old, 25 - 30 g, female Balb/c) and SD rats (4-week-old, 80 - 100g, female SD) were purchased from Hunan Lake Jingda Co., LTD Department. Mice and rats were given free access to food and water. All animal studies were reviewed and approved by the Institutional Animal Care and Use Committee (IACUC) of Renmin Hospital of Wuhan University (Issue No. 20200702). All animal experiments complied with the Guide for Care and Use of Laboratory Animals by the Institute of Laboratory Animal Research.

For the 4T1-luc orthotopic transplantation model, 20 µL of 4T1-luc cells (1× 10^7^ mL^-1^) in PBS were administered i.duc into the 4^th^ right gland of each mouse under anesthesia. Two methods were used to determine the tumor burden. One was to measure the intensity of the bioluminescence signal by *in vivo* imaging, and the other was to determine tumor size by measuring the length and width of the tumors with a digital caliper every 3 days. The tumor volume was calculated as volume (mm^3^) = length × width^2^ × 0.5.

The rats were randomly divided into three groups (n = 10) for the N-methyl-N-nitrosourea (MNU)-induced DCIS model. For the group receiving PBS + laser treatment, on D-7, 40 μL PBS was injected i.duc into the 4^th^ right mammary gland and irradiated at 808 nm for 10 mins; for the PbS/CdS-PEG-Epep group, on D-7, 40 μL PbS/CdS-PEG-Epep was injected i.duc into the 4^th^ right mammary gland; for the PbS/CdS-PEG-Epep + laser group, on D-7, 40 ul PbS/CdS-PEG-Epep was injected i.duc into the 4^th^ right mammary gland and irradiated at 808 nm for 10 mins. On D0 and D7, 20 μL (1.0 mg) MNU was injected i.duc into the 4^th^ right mammary gland of each rat. The time of tumor occurrence was noted, and tumor growth was calculated during the 42-day observation period.

### Histology and electron microscope evaluation

Paraffin-embedded 4 μm tumor tissue sections were deparaffinized, rehydrated, counterstained with hematoxylin and eosin (H&E) (ZSGBBIO), dehydrated with graded alcohol, and cleared in xylene.

For electron microscopy (EM), small pieces of mammary gland tissues (~1 mm^3^) were acquired from the freshly euthanized mice. The samples were fixed, stained, sliced into 70 nm sections, mounted on a grid, and imaged using transmission electron microscopy (TEM) (Tecnai G2, Tecnai G2 F20 S-TWIN, FEI company, USA).

### Immunofluorescence evaluation

For immunofluorescence, primary antibodies against anti-E-cadherin (1:200, Cell Signaling Technology, cat. 14472), α-SMA (1:400, Cell Signaling Technology, cat. 19245), fluorescence-conjugated secondary antibodies (1:500, anti-rabbit, Dylight 488/594, Abbkine, cat. 23220/23430) and anti-fade fluorescence mounting medium with DAPI (ZSGB-BIO) were employed. Sections were viewed and quantified using a Zeiss Axiovert 200 inverted fluorescence microscope and an Olympus AX70 epi-fluorescence microscope.

### Terminal deoxynucleotidyl transferase dUTP nick end labeling assay (TUNEL)

We performed TUNEL staining on cells with the Dead End Fluorometric TUNEL System (Promega). Frozen sections of mammary tissue were processed according to the manufacturer's protocol. Sections were viewed and quantified using a Zeiss Axiovert 200 inverted fluorescence microscope and an Olympus AX70 epi-fluorescence microscope.

### Transcriptomic analysis

RNA extraction, RNA-seq, and transcriptomic data processing were accomplished by BGI Technology Services Co., LTD (Shenzhen, China). RNA reads were mapped, and RPKMs were calculated. Differentially expressed genes (DEG) were defined with FDR corrected p-value ≤ 0.05 and Fold-change ≥2 using the edgeR package. Gene Ontology (GO) and Kyoto Encyclopedia of Genes and Genomes (KEGG) analyses of DEG were implemented by the clusterProfiler R package. Gene Set Enrichment Analysis (GSEA) was performed using GSEA software.

### Flow cytometric analysis

Mammary glands, spleens, and lymph nodes (LNs) were excised from mice and placed on ice. Single-cell suspensions of the mammary gland were obtained by gentleMACS™ dissociator and digestive enzyme (Miltenyi Biotec) according to the manufacturer's instructions. Spleens and LNs were squashed and filtered (70 µm) and cell suspensions were cleared of red blood cells using FACS lysing solution (BD Biosciences). After 2 washes with PBS containing 10% FBS, cells were blocked with anti-CD16/32 Fc blocking antibody (1:25, BD Biosciences, 2.4G2) for 20 minutes. Next, cells were incubated for 30 minutes with antibodies targeting the cell-surface markers anti-CD45 (1:500, eBioscience, 30-F11), anti-CD3 (1:500, Biolegend, 17A2), anti-CD4 (1:500, Biolegend, RM4-5), anti-CD8a (1:200, Biolegend, 53-6.7), anti-CD11b (1:500, Biolegend, M1/70), anti-F4/80 (1:500, Biolegend, BM8), anti-CD86 (1:500, Biolegend, GL-1), anti-CD206 (1:2000, Biolegend, C068C2), anti-CD11c (1:500, Biolegend, N418), anti-Gr-1 (1:500, Biolegend, RB6-8C5), and anti-CD25 (1:500, Biolegend, 3C7). Flow cytometry analysis was performed using a CytoFLEX flow cytometer (Beckman Coulter, Fullerton, CA, USA).

### RNA extraction and real-time qPCR

RNA isolated from mammary glands was prepared using Qiazole/Trizole as the manufacturer's instructions. cDNA was generated on 1.25 µg of RNA using an iScript cDNA synthesis kit (Bio-Rad). Real-time PCR was performed on 4 µL of cDNA product using iTaq Universal SYBR Green Supermix with ROX (Bio-Rad) and the following mouse gene primers: heat shock protein 70 (HSP70), 5′-TGGTGCAGTCCGACATGAAG-3′ (forward) and 5′-GCTGAGAGTCGTTGAAGTAGGC-3′ (reverse); heat shock protein 90 (HSP90), 5′-CTCCATGATCGGGCAGTTTG-3′ (forward) and 5′-CACCACTTCCTTGACCCTC-3′ (reverse); calreticulin (CRT), 5′-ACGAGGAGGAGAGGAAACG-3′ (forward) and 5′-TTAGCTTTTCCCTTCGCAGC-3′ (reverse); high mobility group box 1 (HMGB1), 5′-TCAGCACAGCAACTTCAGG-3′ (forward) and 5′- GTGATTCGCTTGTAGTCCG-3′ (reverse); glyceraldehyde 3-phosphate dehydrogenase (GAPDH), 5′- ATGGGTGTGAACCACGAGA -3′ (forward) and 5′- CAGGGATGATGTTCTGGGCA -3′ (reverse). Real-time PCR was performed on the 7300 Real-Time PCR System (Applied Biosystems).

### Western blot analysis

Mammary glands harvested from treated mice were lysed with RIPA buffer (Beyotime). The protein concentrations were normalized using the BCA protein kit (Beyotime, Cat P0012S), and Western blot analysis was performed. Briefly, the proteins were separated on a 4 - 12% gradient gel (Invitrogen) and transferred to PVDF membranes (Roche). The membranes were blocked with 5% BSA for 1 h and incubated with primary antibodies overnight at 4 °C. Antibodies against the following proteins were used for Western blotting: HSP70 (1:1000, Cell Signaling Technology, cat. 4872), HSP90 (1:1000, Cell Signaling Technology, cat. 4874), CRT (1:1000, Cell Signaling Technology, cat. 12238), HMGB1 (1:1000, Cell Signaling Technology, cat. 6893), and GAPDH (1:5,000, Proteintech, cat. HRP-60004). Signal detection was performed by using an ECL kit (Advansta). The signals were collected and analyzed using an Odyssey system (Li-Cor Biosciences) and Studio software v. 5.2 (LiCor Biosciences).

### Enzyme-linked immunosorbent assay

Mammary gland tissues were collected from mice and homogenized in PBS. The protein concentrations were normalized using a BCA protein kit (Beyotime, Cat P0012S). The tissue levels of IFN-γ (R&D Systems™, cat. MIF00), IL-1β (R&D Systems™, cat. MLB00C), IL-6 (R&D Systems™, cat. DY40605), IL-10 (R&D Systems™, cat. M1000B), and TNF-α (R&D Systems™, cat. MTA00B), were measured with mouse enzyme-linked immunosorbent assay (ELISA) kits according to the manufacturer's protocol.

### Statistical analysis

Data are represented as the mean ± SD as indicated in the figure legends. The Two-tailed Student's t-test was used for two-group comparisons, and one-way ANOVA with Tukey's multiple comparisons was used for multiple comparisons. Categorical data were assessed by chi-square test. Survival curves were graphically displayed using the Kaplan-Meier method and were determined using a log-rank (Mantel-Cox) test. All statistical tests were performed using GraphPad Prism software v. 8.0. Statistical analysis values of *p* < 0.05 were considered significant.

## Results

### Targeted Imaging and Photothermal Ablation of Mammary Ducts Using NIR-IIb Nanoprobes

**Figure 2:**
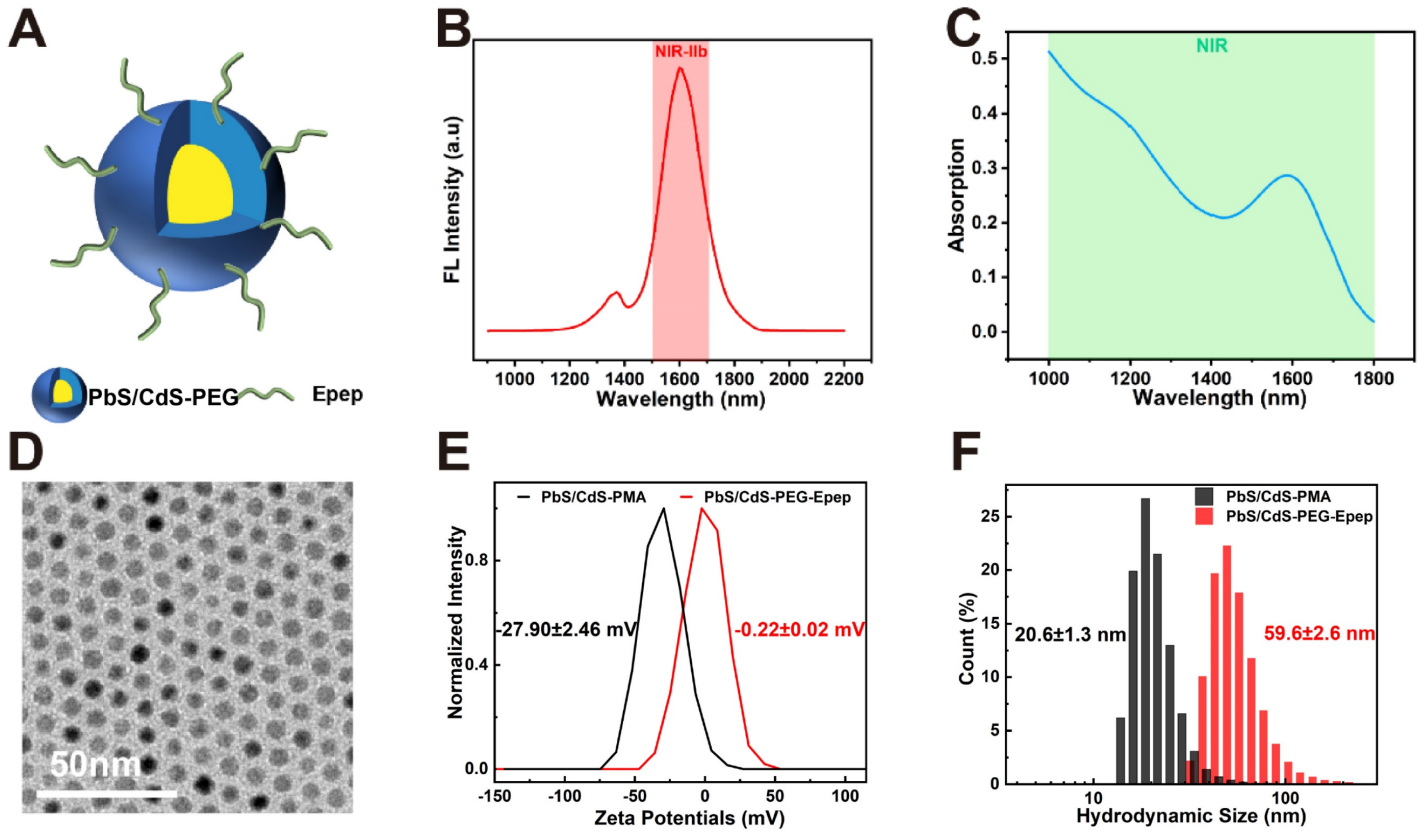
** Characterization of PbS/CdS-PEG-Epep QDs**. **A** Schematic diagram of PbS/CdS-PEG-Epep QDs. **B** PbS/CdS-PEG-Epep QDs emission wavelength is 1600 nm. **C** PbS/CdS-PEG-Epep absorption peak in NIR. **D** Transmission electron microscopy of PbS/CdS-PEG-Epep QDs have regular particle structure and good dispersion. **E** Dynamic light scattering DLS measures the hydrodynamic diameters of PbS/CdS-PMA QDs and PbS/CdS-PEG-Epep QDs. **F** Zeta potential of PbS/CdS-PMA QDs and PbS/CdS-PEG-Epep QDs.

NIR-IIb-emitting PbS/CdS-PEG QDs were synthesized and rendered aqueous by encapsulation with oleyamine-branched poly (acrylic acid) (OPA) as previously described 31. The resulting QDs demonstrated uniformity and excellent dispersibility (Figure 2). *In vivo* nanoprobes were created by surface modification of OPA-coated QDs with a mixture of H-SWELYYPLRANL-NH2 (Epep) peptides and 8-arm PEG-amine molecules using N-(3-(dimethylamino) propyl)-N′-ethylcarbodiimide hydrochloride (EDC) chemistry (Figure 2A-C). Epep peptides were chosen to target E-cadherin, a protein predominantly expressed on ductal epithelial cells, facilitating specific mammary duct targeting 32. Fluorescence emission spectra indicated that PbS/CdS-PEG-Epep QDs emitted intense fluorescence at ~1600 nm (Figure 2B). The broad NIR absorption range (Figure 2C) demonstrated the potential for photothermal conversion in ablation therapy, with a previously reported photothermal conversion efficiency of up to 47.6% 30. Transmission electron microscopy of PbS/CdS-PEG-Epep QDs have regular particle structure and good dispersion (Figure 2D). The PEG molecules were incorporated to enhance *in vivo* stability and biocompatibility of QDs. Dynamic light scattering (DLS) and zeta potential analysis confirmed successful post-modification, increasing hydrodynamic size from 20.6 nm to 59.6 nm and shifting the zeta potential from -27.9 mV to -0.22 mV (Figure 2E&F). Therefore, PbS/CdS-PEG-Epep QDs possessed properties suitable for precise imaging-guided localized treatment and targeted thermal ablation.

**Figure 3:**
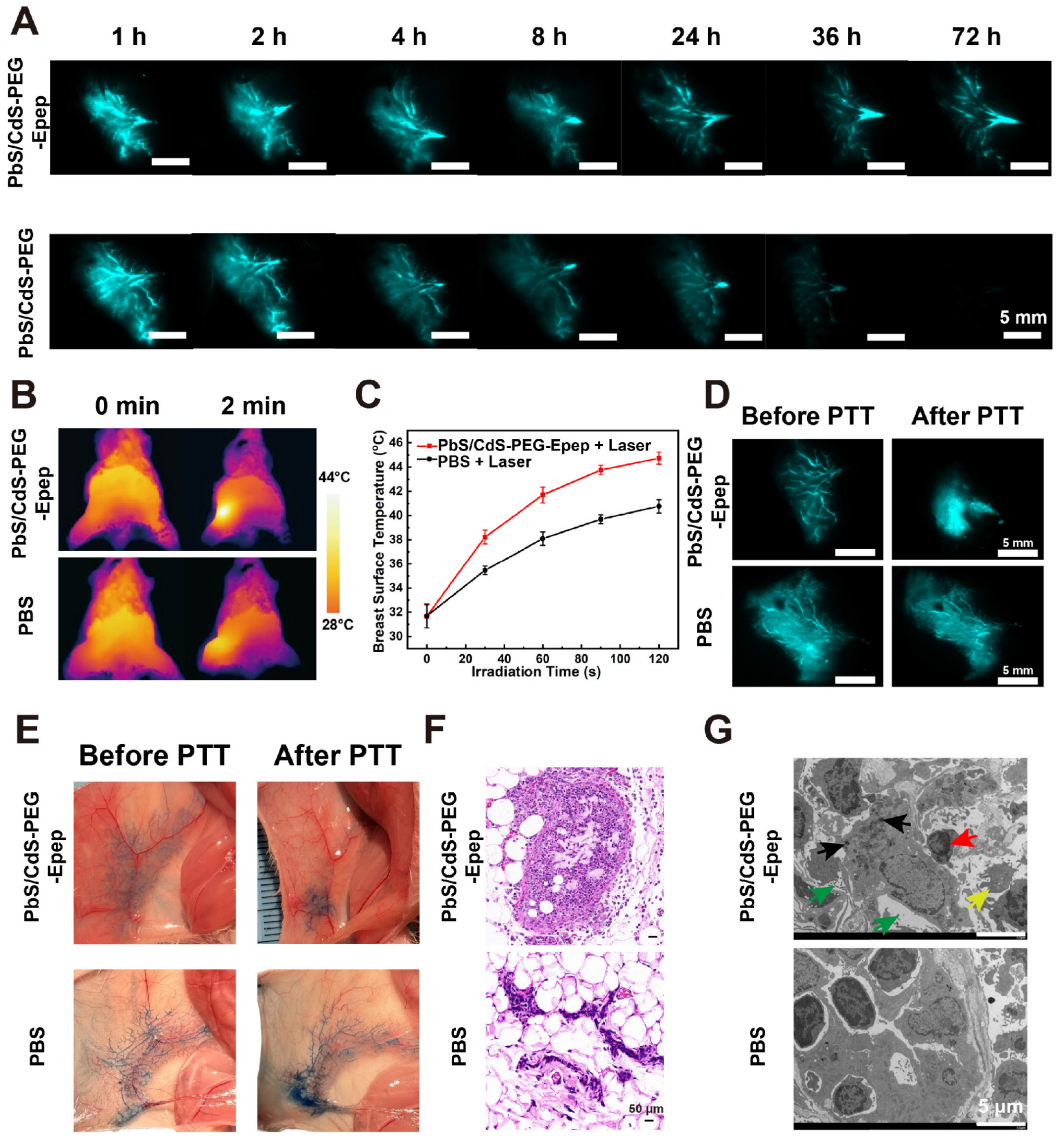
** Targeted mammary gland imaging and intraductal photothermal ablation using NIR-IIb nanoprobes. A** High-magnification fluorescent images depicting the time-course of mammary duct imaging in mice following i.duc injection of PbS/CdS-PEG-Epep QDs and PbS/CdS-PEG QDs within the NIR-IIb window. Images were captured at various time points (1 h, 2 h, 4 h, 8 h, 24 h, 36 h, 72 h) post-injection. (bar = 5 mm. Excitation power density: 0.5 W cm^-2^ from an 808 nm laser.) **B, C** Thermal imaging (**B**) and heating curve (**C**) generated after i.duc injection of PBS and PbS/CdS-PEG-Epep QDs, recorded from 0 to 2 mins during 808 nm laser irradiation. **D** NIR-IIb mammary duct imaging captured before and after photothermal ablation (bar = 5 mm). **E** Observations of mammary duct changes before and after photothermal ablation, visualized through i.duc injection of methylene blue after the mice were sacrificed. **F, G** Morphological alterations in the mammary duct following photothermal ablation, as revealed by H&E staining (**F**, bar = 50 µm) and TEM examination (**G**, bar = 5 µm). After mammary duct ablation, luminal epithelial cells displayed disorganization, loss of microvilli (green arrow), chromosomal pyknosis (red arrow), leading to the formation of numerous autolysosomes (black arrow), and apoptotic bodies (yellow arrow).

To assess the extent of thermal ablation effects accurately, real-time high-resolution imaging of mammary ducts was performed during the local therapy. Before thermal ablation, PbS/CdS-PEG-Epep QDs (30 μL, 1.75 mg mL^-1^) was administered i.duc into the fourth right mammary gland of mice. In the NIR-IIb imaging window, PbS/CdS-PEG-Epep and PbS/CdS-PEG groups exhibited clear visualization of the entire mammary duct with minimal background signals. The signal intensity within the mammary ducts persisted for approximately 24 hours in the PbS/CdS-PEG group, whereas in the targeted mammary ducts (PbS/CdS-PEG-Epep) group, the signal remained strong for at least 72 hours (Figure 3A). These findings underscored the precise and prolonged mammary duct-targeting capability of the PbS/CdS-PEG-Epep QDs.

We proceeded with *in vivo* thermal ablation of mammary ducts. The mammary glands of mice were exposed to an 808 nm laser, with temperature monitoring facilitated by a thermal camera. Figures 3B&C depict the results of this procedure. When the laser power density was set at 0.5 W cm^-2^, mammary gland temperature increased from 31 °C to 44 °C within 2 minutes after administrating PbS/CdS-PEG-Epep QDs *via* i.duc injection. In contrast, the mammary gland temperature in the PBS group only reached 40 °C, indicative of the effective photothermal conversion capability of PbS/CdS-PEG-Epep QDs. Following 2 minutes of irradiation, the mammary gland temperature remained stable between 44-45 °C for 10 minutes, achieving thermal ablation of the mammary ducts. Notably, Figure 3D shows that the characteristic signals of the terminal mammary ducts were no longer detectable in the breast 24 hours after ablation, in contrast to the intact signal in the PBS group. This observation was further supported by direct methylene blue staining of the mammary gland after the i.duc injection (Figure 3E), signifying the disruption of mammary duct integrity, particularly in the terminal ducts.

The mammary glands were examined by H&E staining and TEM 24 hours after laser irradiation to assess histopathological changes in the mammary tissue. Treatment of mammary ducts with PbS/CdS-PEG-Epep + laser resulted in disordered epithelial structures with exfoliated ductal epithelial cells, denatured proteins, lumen swelling, and inflammatory cell infiltration (Figure 3F). These morphological changes were corroborated by TEM. Following mammary duct ablation, the luminal epithelial cells exhibited disorganization, loss of microvilli, and chromosomal pyknosis, leading to the formation of numerous autolysosomes and apoptotic bodies (Figure 3G). After treatment, microscopic examination of the mammary gland provided further evidence of the effective ablation of mammary ducts achieved by PbS/CdS-PEG-Epep QDs.

### Molecular Changes Following Targeted Mammary Duct Ablation with NIR-IIb Nanoprobes

We conducted *in vitro* experiments to confirm the targeting ability of PbS/CdS-PEG-Epep QDs. Ductal epithelial cells (HC11 cells) were incubated with PbS/CdS-PEG-Epep QDs and PbS/CdS-PEG QDs. Subsequently, we imaged the cells in the NIR-IIb fluorescence channels (Supplementary Figure 1A) and quantified the fluorescence intensity (Supplementary Figure 1B). PbS/CdS-PEG-Epep QDs, used to label HC11 cells, exhibited robust fluorescence in the NIR-IIb window. In contrast, the PbS/CdS-PEG group exhibited minimal fluorescence signals. This cell-targeting investigation, demonstrating that PbS/CdS-PEG-Epep QDs could efficiently enter HC11 cells *in vitro*, underscored its strong targeting capabilities and served as a foundation for *in vivo* research.

**Figure 4:**
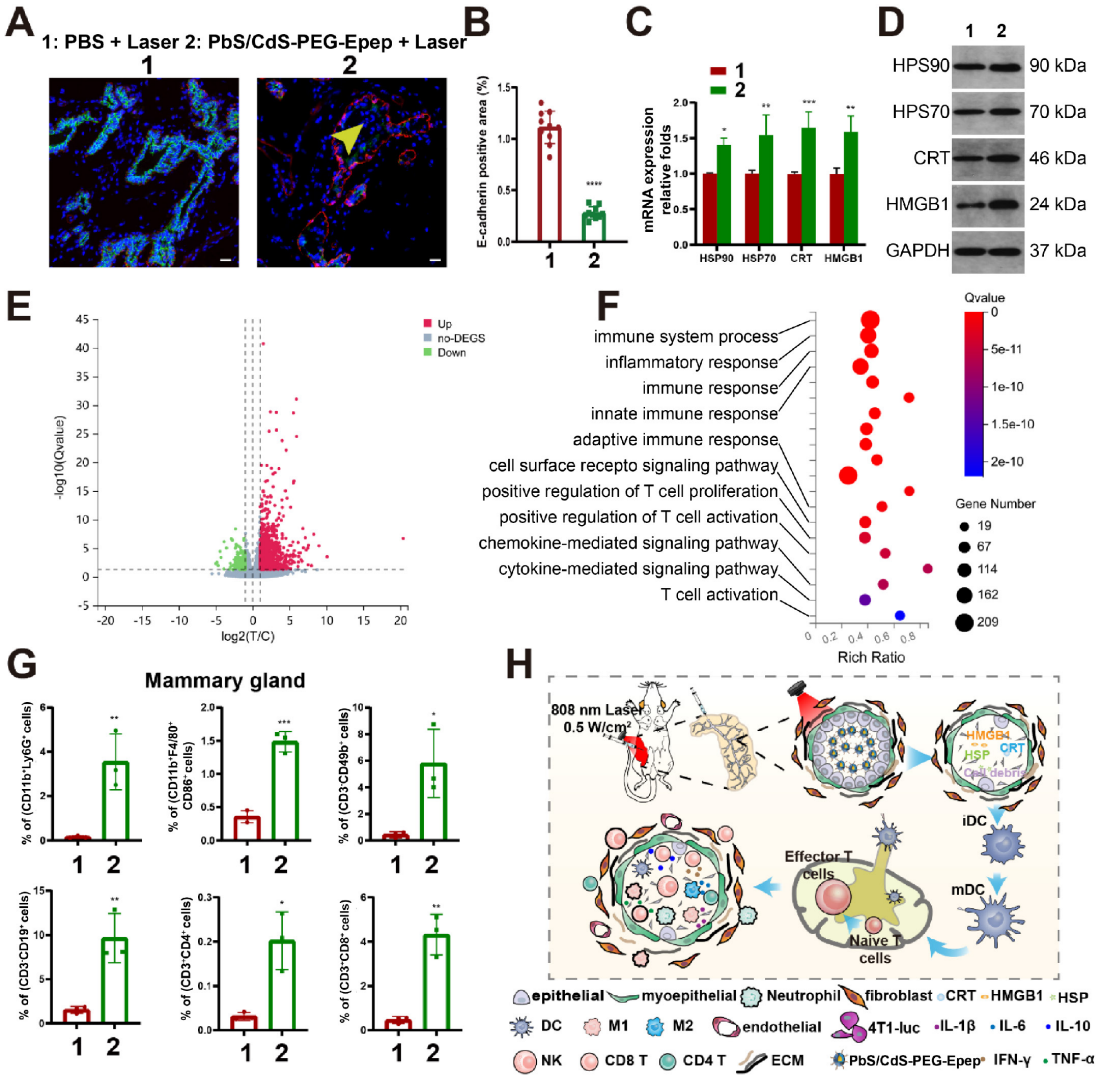
** Molecular alterations and immune responses following targeted mammary duct ablation. A, B** Immunofluorescence and semi-quantitative fluorescence intensity analysis of mammary tissues, E-cadherin (green), exfoliated epithelial cells (yellow arrows), α-SMA (red) and DAPI (blue), bar = 50 µm. **C, D** mRNA and protein levels of HSP70, HSP90, CRT, and HMGB1 in the mammary gland 24 hours after ductal ablation. **E** Volcano map of differentially expressed genes in the mammary gland seven days after duct ablation. **F** GO terms enrichment in immune-related function. **G** Flow cytometry analysis of neutrophils (CD11b^+^Ly6G^+^), M1 macrophages (CD11b^+^F4/80^+^CD86^+^), natural killer cells (CD3^-^CD49^+^), B lymphocytes (CD3^-^CD19^+^), CD4 T cells (CD3^+^CD4^+^), and CD8 T cells (CD3^+^CD8^+^) in mammary microenvironment. **H** Schematic diagram of immune activation caused by mammary ductal ablation by PbS/CdS-PEG-Epep QDs. The data were expressed as means ± SD, ^*^p < 0.05,^ **^p < 0.01, ^***^p < 0.001, ^****^p < 0.0001.

We examined key markers to gain insights into the molecular changes after mammary gland ablation. In the PbS/CdS-PEG-Epep group, the luminal epithelial marker (E-cadherin) exhibited a significant reduction (Figure 4A&B), as revealed by fluorescence duplex staining of E-cadherin and α-SMA (a myoepithelial marker). Additionally, the TUNEL assay indicated a substantial increase in apoptosis (Supplementary Figure 2A). These findings collectively suggested the loss of epithelial-specific signals and the induction of apoptosis in mammary glands following treatment with NIR-IIb nanoprobes.

### Critical Role of Immune Response in Tumor Ablation with NIR-IIb Nanoprobe

Previous research has demonstrated the induction of immunogenic cellular death (ICD) following thermal ablation 19-23. Ablated tumor cells could transition from a non-immunogenic state to an immunogenic one, characterized by the release of secretory damage-associated molecular patterns (DAMPs), including HSP70, HSP90, CRT, and HMGB1. Therefore, we explored the immune responses triggered by thermal ablation within the mammary ductal microenvironment (Figure 4, Supplementary Figures 2&3). Employing RT-qPCR (Figure 4C) and Western blot analysis (Figure 4D), we observed a significant upregulation of mRNA and protein levels of HSP70, HSP90, CRT, and HMGB1 in the PbS/CdS-PEG-Epep + laser group compared to the control group (PBS + laser).

Subsequently, we conducted a thorough examination of immunological changes within the mammary ductal microenvironment in our mouse model by performing RNA-seq analysis, ELISA, and flow cytometry to assess alterations in gene expression, cytokine and chemokine secretion, and immune cell infiltration in the control group versus the PbS/CdS-PEG-Epep + laser group. RNA-seq analysis revealed that, compared to the control group, 1,974 genes were upregulated while 1,162 genes were downregulated in the mammary ductal microenvironment of the PbS/CdS-PEG-Epep + laser group (Figure 4E and Supplementary Figure 2D). Furthermore, gene set enrichment analysis (GSEA) highlighted that differentially expressed genes were predominantly enriched in the antigen presentation pathway, with a normalized enrichment score (NSE) of 2.57 (Supplementary Figure 2C). Kyoto Encyclopedia of Genes and Genomes (KEGG) analysis illustrated the activation of immune system-related pathways (Figure 4F). Additionally, ELISA assays indicated a significant increase in cytokine levels, including IL-1β, IL-6, IL-10, IFN-γ, and TNF-α, compared to the control group (Supplementary Figure 2E).

We conducted flow cytometry to assess immune cell infiltration within the mammary microenvironment. Compared to the control group, there was a significant increase in the proportion of immune cells, including neutrophils (CD11b^+^Ly6G^+^), M1 macrophages (CD11b^+^F4/80^+^CD86^+^), natural killer cells (CD3^-^CD49^+^), B lymphocytes (CD3^-^CD19^+^), CD4 T cells (CD3^+^CD4^+^), and CD8 T cells (CD3^+^CD8^+^) in the PbS/CdS-PEG-Epep + laser group (Figure 4G). Notably, the proportion of macrophages, particularly the M1 subtype, were significantly increased, enhancing their antigen presentation capacity. These findings strongly suggested the immunological activation of the mammary microenvironment post-thermal ablation.

Furthermore, there was a statistically significant increase in the number of mature dendritic cells (CD11b^+^CD11c^+^MHCII^+^CD86^+^) and CD8 T cells (CD3^+^CD8^+)^ in the inguinal lymph nodes of the PbS/CdS-PEG-Epep + laser group compared to the control group (Supplementary Figure 2F). In conclusion, mammary duct ablation induced DAMPs, enhanced antigen presentation, facilitated dendritic cell maturation, and promoted immune cell infiltration and functional activation, likely triggering immune responses (Figure 4H). We also examined the immune response elicited by PbS/CdS-PEG-Epep QDs as an additional control. DAMPs and immune cell infiltration exhibited no statistically significant differences among three groups (PBS, PBS + Laser, and PbS/CdS-PEG-Epep), indicating that the nanoparticles alone did not induce substantial immune enhancement in the absence of NIR laser-induced thermal effects (Supplementary Figures 2G&H).

### *In Situ* Mammary Duct Ablation for Early-Stage TNBC Treatment

**Figure 5:**
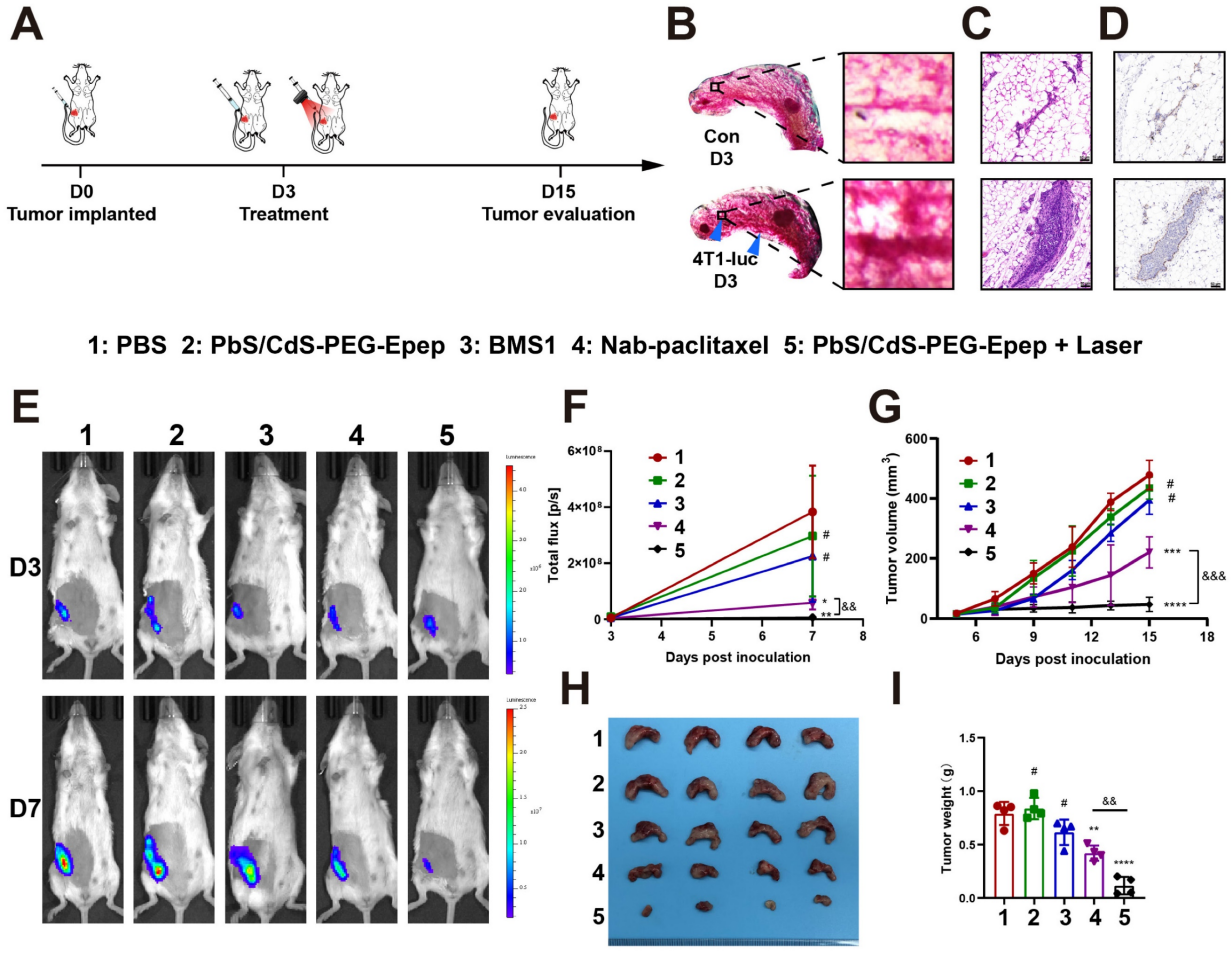
** Mammary duct ablation *in situ* for early-stage TNBC treatment. A** Schematic diagram of the design of ductal tumor ablation in 4T1-luc mouse model. **B-D** Early-stage TNBC in mice was confirmed by whole-mount mammary gland staining (**B**), integrity of the ductal epithelium by H&E staining (**C**, bar = 50 μm) and immunofluorescence staining for α-SMA (myoepithelial marker) staining (**D**, bar = 50 μm). **E, F** *In vivo* imaging and fluorescence signal analysis of different treatment groups on day 3 and 7 after tumor inoculation (n = 4). **G** Growth curve of 4T1-luc transplanted tumors in different treatment groups after tumor inoculation. **H, I** Images of tumor (**H**) and statistical analysis of tumor weights (**I**). The data are expressed as the means ± SD, ^#^*p* > 0.05, ^*^p < 0.05, ^**^p < 0.01, ^***^p < 0.001, ^****^p < 0.0001 *vs* PBS group; ^&&^*p* < 0.01, ^&&&^*p* < 0.001.

We validated the antitumor effects of mammary duct ablation on BC treatment and prevention in various animal models (Supplementary Figure 4). These models included early-stage TNBC treatment, neoadjuvant TNBC, and BC prevention in an MNU-induced DCIS model. We assessed the anti-tumor effects of mammary duct ablation in early TNBC (Figure 5A). 4T1-luc cells were intraductally injected into the mammary gland to create the *in situ* TNBC model. Three days later, the tumor remained predominantly confined within the mammary duct, as confirmed by morphological evaluations, observable thickened ductal structures in whole-mounts of the mammary gland (Figure 5B), preservation of ductal integrity as evidenced by H&E staining (Figure 5C), a-SMA immunohistochemical staining (Figure 5D), and the detection of weak fluorescence signals by *in vivo* biotin fluorescence imaging (Figure 5E). These collective findings underscored the relevance of our model system for investigating early TNBC.

Next, 20 mice bearing intraductally implanted 4T1-luc TNBC were randomly assigned to five groups, i.duc treated with PBS (control), PbS/CdS-PEG-Epep, PD-1/PD-L1 inhibitor (BMS1), Nab-paclitaxel (chemotherapy), and PbS/CdS-PEG-Epep + Laser (ablation). After seven days, *in vivo* imaging revealed a significantly lower luminescence intensity in the PbS/CdS-PEG-Epep + Laser group than in the other four groups (Figures 5E&F). Furthermore, continuous monitoring of the tumor size demonstrated that tumors in the PbS/CdS-PEG-Epep + Laser (ablation) group remained consistently smaller, while those in the other four groups gradually increased in size (Figure 5G). This observation was reaffirmed during necropsy on day 15, where the PbS/CdS-PEG-Epep + Laser (ablation) group exhibited the smallest tumors (Figure 5H) in size and weight (Figure 5I). Further analysis revealed that the tumor burden in the PbS/CdS-PEG-Epep + Laser group was reduced by 85.4% and 72.6% compared to the control group and the Nab-paclitaxel group, respectively, highlighting the highly significant anti-tumor effects of PbS/CdS-PEG-Epep + Laser in mammary duct ablation for early TNBC.

### *In Situ* Enhanced Effects of Mammary Duct Ablation Following Neoadjuvant TNBC Treatment

**Figure 6:**
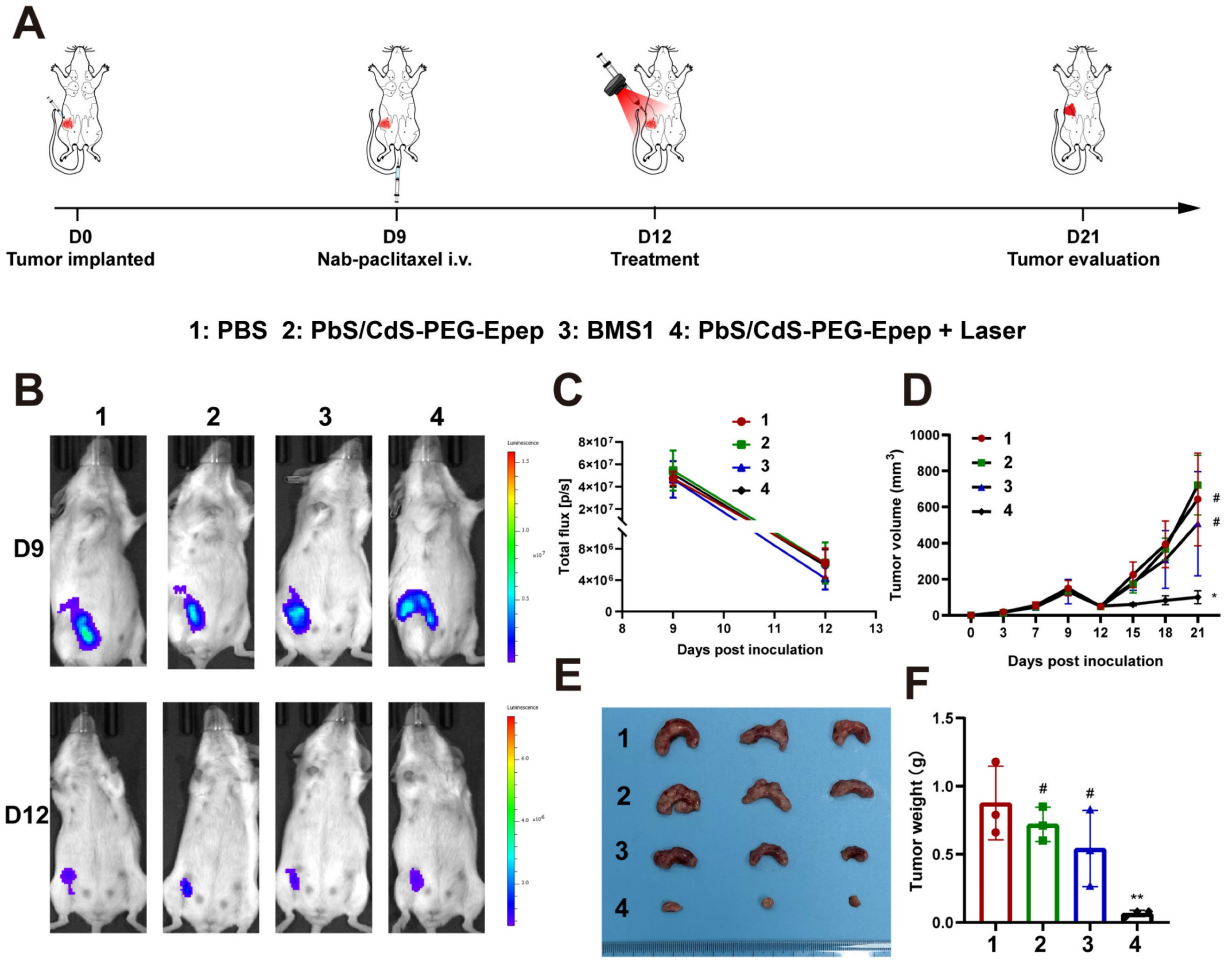
** Mammary duct ablation enhanced neoadjuvant effect of nab-paclitaxel in TNBC. A** Schematic diagram of mammary duct ablation mimicking TNBC neoadjuvant treatment. On the 9^th^ day of tumor growth, clinical neoadjuvant treatment is simulated by intravenous administration of nab-paclitaxel. After tumor reduction, various treatments are started on the 12^th^ day, and their antitumor efficacy is compared. **B, C** *In vivo* imaging and fluorescence signal curve of PBS, PbS/CdS-PEG-Epep, BMS1, and PbS/CdS-PEG-Epep + Laser groups on days 9 and 12 after tumor inoculation (n = 3). **D** Growth curve of 4T1-luc transplanted tumor in different treatment groups. **E, F** Image of tumor removed from mice 21 days after tumor inoculation and statistical analysis of tumor weights (**F**). Data are expressed as the means ± SD, ^#^*p* > 0.05, ^*^p < 0.05, ^**^p < 0.01.

Neoadjuvant therapy is a widely adopted treatment approach for BC characterized by larger tumor size or lymph node metastasis. Here, we employed a 4T1-luc TNBC mouse model to assess the impact of mammary duct ablation on anti-tumor effects in the neoadjuvant context. 4T1-luc TNBC cells were intraductally injected and allowed to grow until day 9 to reach an approximately 120 mm³ tumor size. At this juncture, tumor cells had minimally invaded the parenchyma. Subsequently, Nab-paclitaxel was intravenously administered to the mice at a dose of 260 mg m⁻² per mouse. On day 12, the mice were randomly assigned to four groups and treated with PBS, PbS/CdS-PEG-Epep, BMS1, or PbS/CdS-PEG-Epep + Laser (Figure 6A).

Following Nab-paclitaxel treatment on day 9, there was a remarkable reduction of approximately 90% in tumor size by day 12 (Figure 6B-D). Subsequent treatments resulted in varying tumor responses: in the PbS/CdS-PEG-Epep + Laser group, tumors displayed no significant change in size, while tumors in the other three groups exhibited size increases (Figure 6D). These observations were further validated by comparing the weights of tumors among groups after the mice were sacrificed on day 21 (Figures 6E & F). Notably, the tumor weight in the control group was 13 times greater than that in the PbS/CdS-PEG-Epep + Laser group (^*^p < 0.05).

### *In Situ* Mammary Duct Ablation for BC Prevention

BC prevention measures are of paramount importance for women with a heightened risk of developing BC due to inherited or acquired susceptibilities. We conducted a comprehensive investigation to assess the viability of our duct ablation approach for BC prevention. Initially, we ablated the mammary ducts of mice using the PbS/CdS-PEG-Epep QDs in combination with laser exposure (Supplementary Figure 5A). Subsequently, 4T1-luc cells were intraductally injected after a 7-day interval. Tumor growth was monitored for 18 days. The results revealed that the treatment inhibited *in situ* tumor growth (Supplementary Figure 5B-F) and reduced the number of lung metastases (Supplementary Figure 5G-I). In this model, while the aggressive growth of 4T1-luc cells within the mammary ducts was inhibited, it was not entirely prevented, possibly due to the 4T1-luc cell growth in the stromal tissue of the mammary fat pad in the presence of damaged ducts.

**Figure 7:**
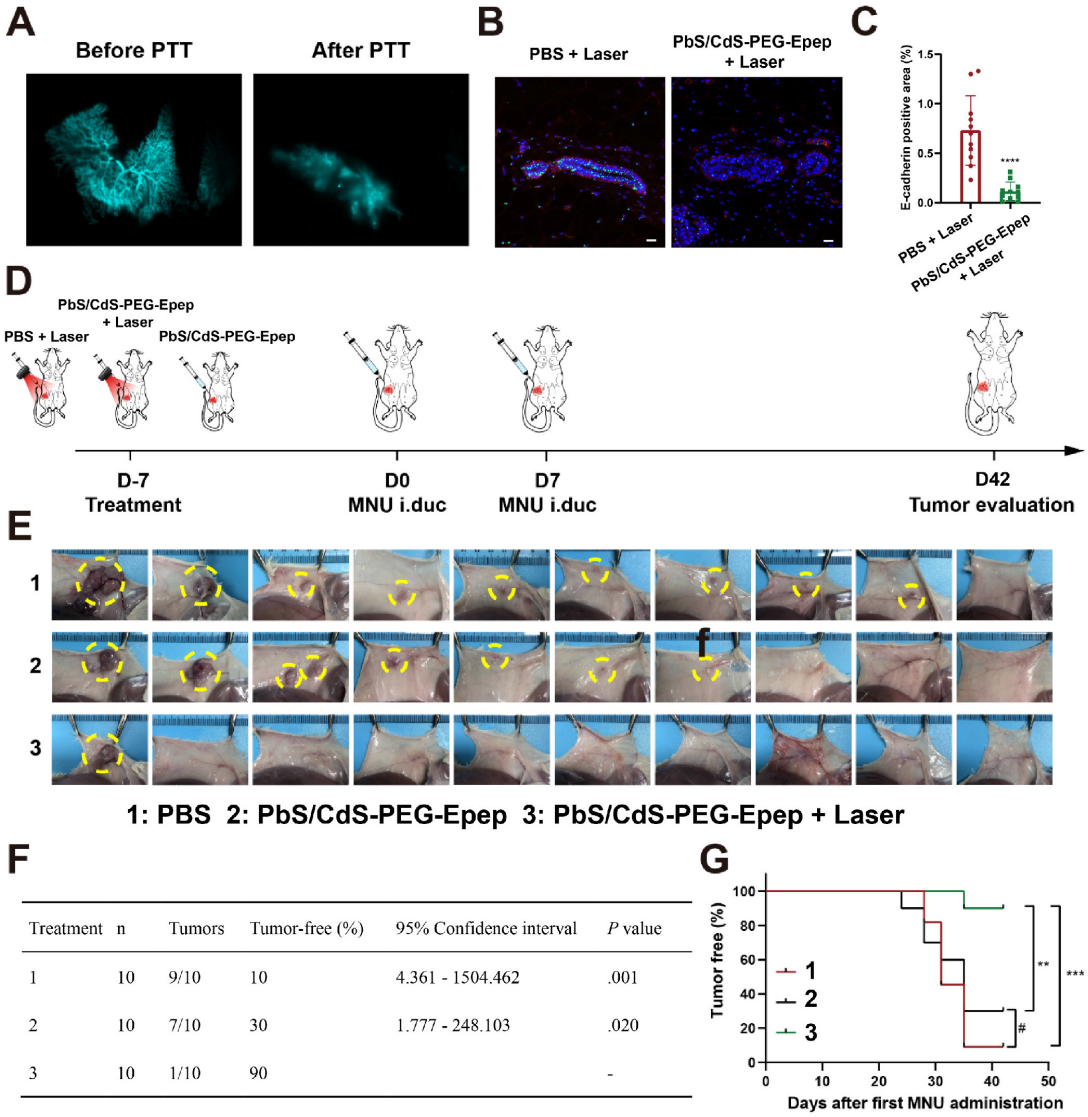
** Mammary duct ablation in rat and BC prevention in DCIS models. A** NIR-Ⅱb based mammary duct imaging before and after PbS/CdS-PEG-Epep photothermal ablation in rats. **B, C** Immunofluorescence and semiquantitative fluorescence intensity analysis of E-cadherin expression in mammary tissues (bar = 50 µm), data are expressed as the means ± SD, ^****^p < 0.0001. **D** Schematic diagram of the experimental design. **E** Tumors in the mammary gland are shown on day 42 after different treatments, and the tumor is circled by the yellow dotted line.** F** Incidence of tumors in different treatment groups. **G** Tumor-free survival curve of rats in different treatment groups. Statistical significance was determined using the chi-square test, ^#^*p* > 0.05, ^**^p < 0.01, ^***^p < 0.001.

We investigated the feasibility of mammary duct ablation for DCIS prevention in a clinically relevant MNU-induced DCIS model in rats. Tumors induced by the chemical carcinogen MNU typically exhibited estrogen receptor-positive characteristics and displayed less aggressive biological behaviors, consistent with previous studies 15,16,18. In our recent study, we modified this model by intraductally injecting MNU, resulting in consistent and predictable tumor locations, sizes, and appearance times 33. We initially standardized mammary duct imaging and photothermal ablation using the PbS/CdS-PEG-Epep QDs in this autochthonous DCIS rat model. Effective destruction of the mammary terminal ducts was confirmed after ablation (Figure 7A) and further validated by H&E staining (Supplementary Figure 6A), TEM (Supplementary Figure 6B), and immunofluorescence staining (Figures 7B&C, and Supplementary Figures 6C&D) of the mammary gland sections.

Subsequently, we evaluated the impact of mammary duct ablation on MNU-induced tumorigenesis in rats. Thirty female SD rats were randomly divided into three groups (PBS + Laser, PbS/CdS-PEG-Epep, and PbS/CdS-PEG-Epep + Laser) (Figure 7D). Seven days later, MNU was intraductally administered once a week for two weeks. The rats were then monitored for tumor formation over six weeks. One single tumor developed (1/10 rats, 10%) with the longest latency in the PbS/CdS-PEG-Epep + Laser group in contrast to the tumor formation incidence in the control group (PBS + Laser: 9/10, and PbS/CdS-PEG-Epep: 7/10) (Figures 7E&F) (*p* < 0.05).

Furthermore, the tumor-free survival analysis showed significant differences in the PbS/CdS-PEG-Epep + Laser group compared to the other two groups (*p* < 0.01) (Figure 7G). Throughout the experiment, body weight in the three groups displayed no statistically significant differences (Supplementary Figure 6E).

### Toxicity and Safety Assessment of Intraductally Administered PbS/CdS-PEG-Epep QDs

**Figure 8:**
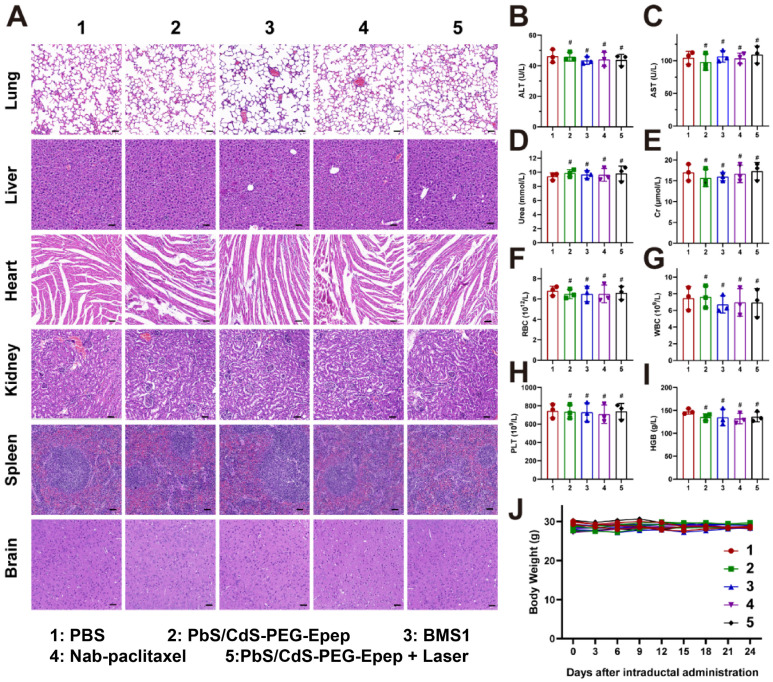
** Toxicity and safety assessment of PbS/CdS-PEG-Epep QDs by i.duc administration to mice. A** H&E staining images of the lung, liver, heart, kidney, spleen and brain after intraductal injection of PBS, PbS/CdS-PEG-Epep, BMS1, Nab-paclitaxel, PbS/CdS-PEG-Epep + Laser for 24 days (n = 3), bar = 50µm. **B-I** alanine aminotransferase (ALT), aspartate aminotransferase (AST), Urea, creatinine (Cr), white blood cell (WBC), red blood cell (RBC), platelets (PLT), hemoglobin (HGB). **J** Body weight of mice after different treatments (n = 3). The data were expressed as means ± SD, ^#^*p* > 0.05.

The toxicity and safety of the intraductally administered PbS/CdS-PEG-Epep QDs were comprehensively assessed following the methodology outlined in our previous publications 27. No discernible structural damage or indications of inflammation were detected in major organs, including the lung, liver, heart, spleen, kidney, and brain, across all treatment groups (Figure 8A). Furthermore, biochemical and hematological analyses, including alanine aminotransferase (ALT), aspartate aminotransferase (AST), urea, creatinine (Cr), white blood cell (WBC), red blood cell (RBC), and platelet (PLT) counts, and hemoglobin level (HGB), revealed no toxic effects on liver and renal functions, or blood parameters in any of the treatment groups (Figures 8B-I). There was no statistically significant difference in the body weight of mice among the groups (Figure 8J).

An additional group of five rats that underwent PbS/CdS-PEG-Epep + Laser mammary duct ablation was monitored for 52 weeks to assess the long-term effectiveness of tumor prevention. No tumors developed during this period, and no long-term systemic adverse reactions were observed over 52 weeks (Supplementary Figure 7).

## Discussion

Surgery of solid cancers has shifted toward more precise and minimally invasive treatment strategies, driven by advancements in fluorescence imaging-guided nanotechnology and systemic therapies 34-36. This trend is also reflected in the exploration of breast surgery de-escalation and omission over the past decades 2-10. A substantial portion of early BC patients, including those with DCIS, microinvasion, and those with favorable responses to neoadjuvant therapy, are potential candidates for minimally invasive local treatment. Our approach of intraductal therapy *via* the natural opening of the nipple has emerged as a non-invasive method for early BC treatment, substantiated by our prior preclinical and clinical investigations 7, 15-18.

Leveraging the potential of functional nanomaterial-based photothermal therapy (PTT) has offered promising prospects for minimally invasive cancer therapy. This approach allows for precise targeting of cancerous lesions while minimizing harm to healthy tissues and augments systemic therapy 19, 37-39. The utilization of the NIR-II imaging window, known for its high resolution and deep tissue penetration capabilities, has become a valuable asset in imaging-guided therapies for precision medicine 19-30. In this study, the development of NIR-IIb nanoprobes by conjugating PbS/CdS-PEG QDs with Epep polypeptide allowed for optimal photothermal effects and precise targeting of mammary ductal epithelial cells. These nanoprobes were administered intraductally to access and efficiently target mammary ductal epithelial cells. The high contrast imaging facilitated by NIR-IIb enabled non-invasive resolution of the mammary duct structure, thus enabling accurate thermal ablation for localized BC therapy.

A significant aspect of our study is that the entire mammary gland was imaged *in vivo* for the first time and ablated in various animal models. The application of low irradiation density, capitalizing on the high photothermal conversion efficiency of NIR-IIb PbS/CdS-PEG QDs, rapidly elevated the temperature of the mammary gland to around 44°C. Importantly, the thermal ablation primarily targeted the terminal ducts, sparing the skin from injury and preventing systemic inflammatory responses. It is of note that the terminal duct ablation potentially affected tumorigenesis at this site.

It is well established that tissues exposed to temperatures between 42-46°C for 10 minutes can lead to cell necrosis, immune stimulation, and the induction of ICD characterized by the expression of secretory DAMPs, such as CRT, ATP, HMGB1, HSP90, and HSP70. These DAMPs facilitate the uptake of cell debris by dendritic cells (DCs), resulting in the initiation of adaptive immune responses 19-23. In our study, mammary duct photothermal ablation caused localized damage to target lesions and also induced ICD within the microenvironment, leading to immune responses evidenced by the upregulation of endogenous molecules (CRT, HSP70, HSP90, and HMGB1) and increased levels of immune cytokines (IL-1β, IL-6, IL-10, IFN-γ, and TNF-α). This cascade of events led to immune cell infiltration, including neutrophils, natural killer cells, B cells, and M1 macrophages, and maturation of antigen-presenting cells (DCs). The infiltrating immune cells migrated to lymph nodes, promoting T cell proliferation and activation, ultimately creating an immune-activated microenvironment. Therefore, NIR-IIb imaging-guided mammary duct ablation through the intraductal route may lead to potent antitumor effects by accurately ablating terminal ducts and activating immune responses.

The antitumor effects of mammary duct ablation were further assessed in BC models, with a particular focus on TNBC, known for its invasiveness and heterogeneity 2, 40. We established TNBC models by intraductal injection of 4T1-luc cells into the mammary ducts of mice. Our results demonstrated a significantly higher efficacy of tumor inhibition with duct ablation than traditional therapy for early TNBC treatment. Additionally, the effectiveness of tumor ablation in a neoadjuvant treatment model of TNBC involving 4T1-luc xenograft tumors was evident. Therefore, NIR-IIb imaging-guided mammary duct ablation through intraductal therapy provides an accurate and efficient non-invasive approach for early TNBC treatment.

BC prevention remains a critical area of exploration, particularly for individuals at high risk of developing BC. Although tumor prevention was not achieved in the rapidly growing 4T1-luc model, we observed significantly reduced tumor growth and fewer lung metastases after ablation. We employed a clinically relevant MNU-induced DCIS rat model to validate the feasibility of mammary duct ablation for DCIS prevention. This model is characterized by luminal BC subtype features and less aggressive behavior, supported by previous studies 15, 16, 18, 33. Targeted mammary duct ablation effectively and non-invasively prevented tumor development and growth *in situ* in most rats. Significantly, accurate duct destruction did not alter the shape of the mammary gland and exhibited no noticeable long-term systemic adverse reactions, offering a promising non-invasive approach for DCIS treatment and prevention.

While our study has made substantial contributions, several areas merit further investigation. First, the complex mechanisms underlying the antitumor effects of mammary duct ablation require in-depth exploration, particularly in clinical studies. Second, the potential of mammary ablation as an "*in situ* vaccine," capable of enhancing responses when combined with other therapeutic modalities, such as immunotherapy, chemotherapy, gene therapy, and radiotherapy, should be rigorously assessed. Third, the design of nanoprobes should be refined to enhance accuracy and efficiency and reduce toxicity. Examples include the development of probes specific to certain BC subtypes and multi-modal probes effective against both the duct and the microenvironment. Lastly, while systemic toxicity was not observed in animal models, the long-term effects of ablation should be further evaluated, particularly for BC prevention in clinical studies.

In summary, our study successfully introduced intraductally administered NIR-II QDs nanoprobe for the first time, enabling targeted mammary duct imaging *in vivo*. Significantly, the mammary ducts could be accurately and efficiently ablated *in situ* by NIR-II photothermal effects. Targeted mammary duct ablation demonstrated substantial efficacy in BC treatment and prevention, driven by localized effects and activated immune response. This approach provides a novel, precise, efficient, and non-invasive strategy for local BC therapy. It also opens new avenues for further exploring the application of this technique in various BC subtypes and other aspects of cancer management, enhancing our understanding of BC prevention and treatment paradigms.

## Supplementary Material

Supplementary figures.

## Abbreviations

α-SMA: alpha smooth muscle actin
CRT: calreticulin
DAPI: 4′,6-diamidino-2-phenylindole
DCIS: ductal carcinoma *in situ*
GAPDH: glyceraldehyde 3-phosphate dehydrogenase
GSEA: gene set enrichment analysis
H&E: hematoxylin and eosin
HMGB1: high mobility group box 1
HSP: heat shock protein
HSP70: heat shock protein 70
HSP90: heat shock protein 90
MNU: N-methyl-N-nitrosourea
Nab-paclitaxel: nanoparticle albumin-bound paclitaxel
NIR-IIb: near-infrared IIb
PTT: photothermal therapy
TEM: transmission electron microscope
TNBC: triple-negative breast cancer
TUNEL: terminal deoxynucleotidyl transferase dUTP nick end labeling
