# Supplementary material for: Intraductal photothermal ablation: a noninvasive approach for early breast cancer treatment and prevention
Source: Theranostics. 2024 Jul 1;14(10):3997–4013. doi: 10.7150/thno.97968 (PMC11234271; doi:10.7150/thno.97968)
Supplement: Supplementary file 1 — Supplementary figures. [file thnov14p3997s1.pdf]

Supplementary Information

Intraductal photothermal ablation: a noninvasive approach for early breast cancer treatment and prevention

Jianhua Liu[1, 2#], Biao Huang[3#], Yan Rao[4#], Liantao Guo[1], Cheguo Cai[5], Dongcheng Gao[1], Deguang Kong[1], Guannan Wang[6], Yao Xiong[1], Ran Cui[3*], Mingxi Zhang[7*], Chuang Chen[1*]

[1] Department of Breast and Thyroid Surgery, Renmin Hospital of Wuhan University, Wuhan, Hubei 430060, PR China

[2] Department of Breast Surgery, Hubei Cancer Hospital, Tongji Medical College, Huazhong University of Science and Technology, Hubei Provincial Clinical Research Center for Breast Cancer, Wuhan Clinical Research Center for Breast Cancer. No.116 Zhuo Daoquan South Road, Wuhan, Hubei 430079, PR China

[3] College of Chemistry and Molecular Sciences, Wuhan University, 430072 Wuhan, PR China

[4] Animal Biosafety Level III Laboratory at the Center for Animal Experiment, Wuhan University School of Medicine, Wuhan, 430071, PR China

[5] Department of Thyroid and Breast Surgery, Frontier Science Center for Immunology and Metabolism, Medical Research Institute, Zhongnan Hospital of Wuhan University, Wuhan University, Wuhan, 430071, PR China

[6] Lombardi Comprehensive Cancer Center, Georgetown University, 3970 Reservoir Rd NW, New Research Building, Room E204, Washington, D.C. 20007, USA

[7] State Key Laboratory of Advanced Technology for Materials Synthesis and Processing, Wuhan University of Technology, 430070 Wuhan, PR China.

[#]These authors contributed equally to this work.

[*]Corresponding author: Chuang Chen, M. D., Department of Breast and Thyroid Surgery, Renmin Hospital of Wuhan University, No 238 Jiefang Road, Wuchang District, Wuhan, Hubei 430060, PR China. E-mail: chenc2469@whu.edu.cn.

Ran Cui, College of Chemistry and Molecular Sciences, Wuhan University, 430072 Wuhan, China. E-mail: cuiran@whu.edu.cn.
Mingxi Zhang, State Key Laboratory of Advanced Technology for Materials Synthesis and Processing, Wuhan University of Technology, 430070 Wuhan, China. E-mail: mxzhang@whut.edu.cn.

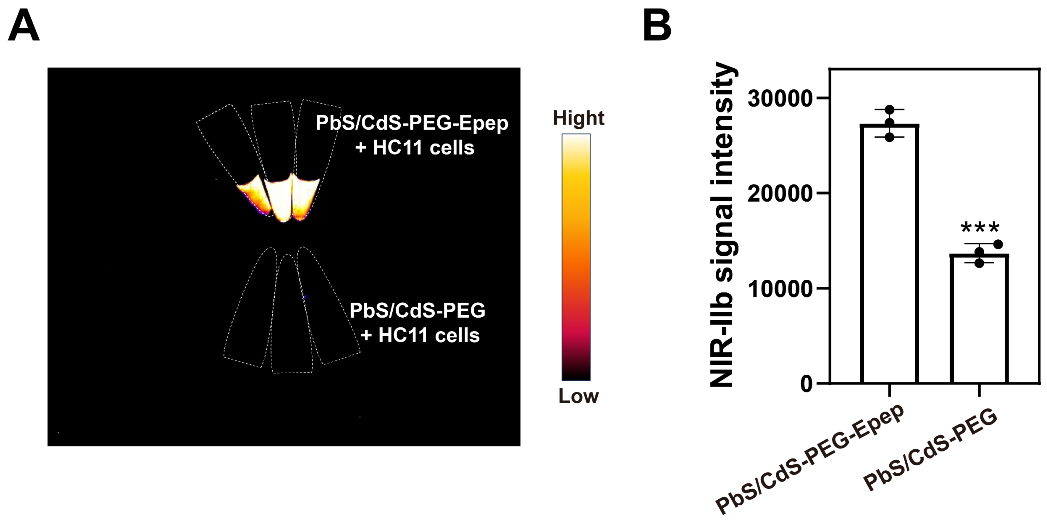

Supplementary Figure 1 Targeting of PbS/CdS-PEG-Epep QDs *in vitro*. A NIR-II fluorescence images for HC11 cells after being incubated with PbS/CdS-PEG-Epep QDs and PbS/CdS-PEG QDs, respectively. 808 nm excitation and 980 nm long pass plus 1500 nm long pass emission filters for NIR-IIb fluorescent channels (n = 3). **B** NIR-II signal intensity of PbS/CdS-PEG-Epep QDs and PbS/CdS-PEG QDs, data were expressed as means ± SD, $^{***}p < 0.001$.

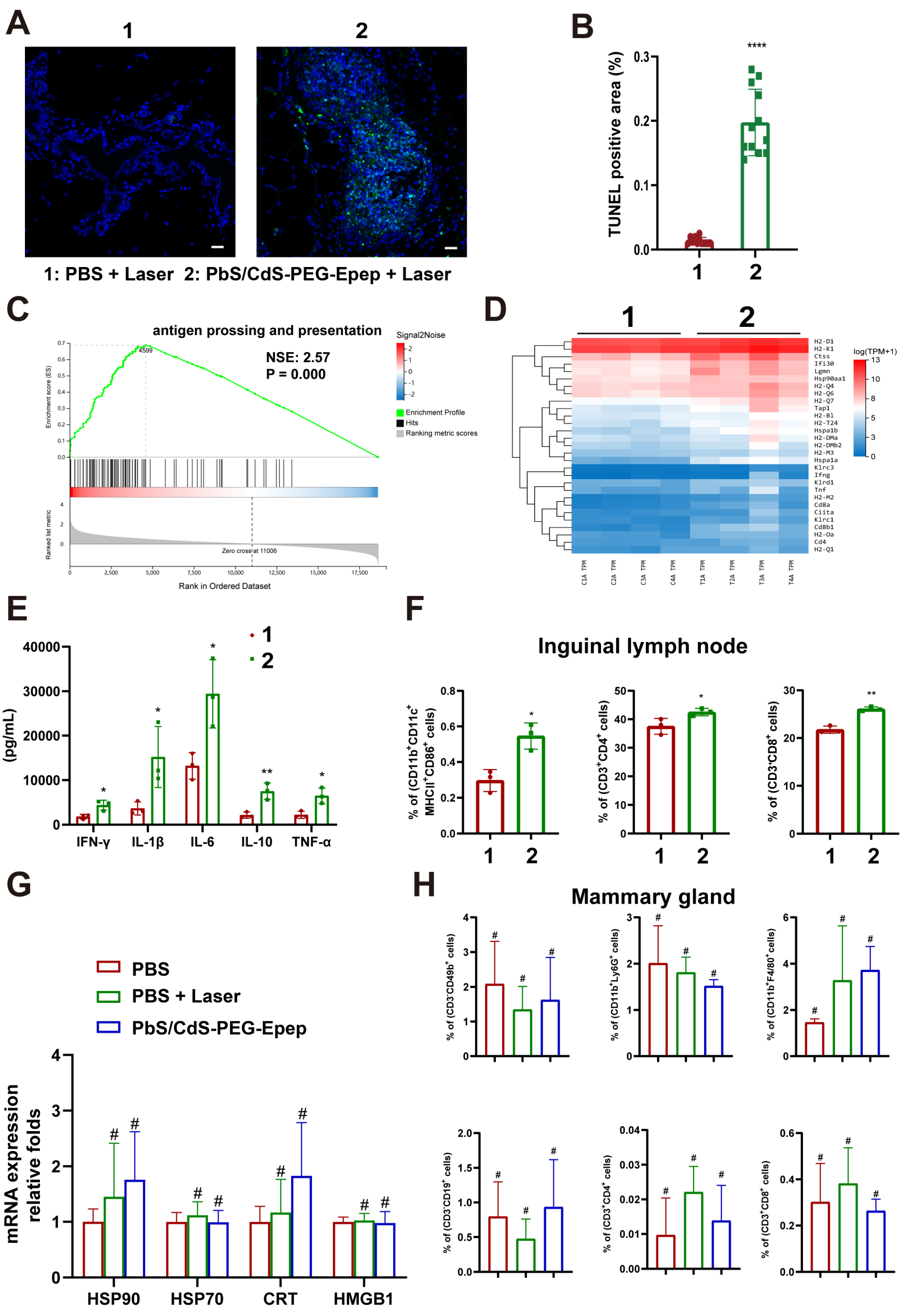

Supplementary Figure 2 Immune changes after mammary duct ablation A, B Immunofluorescence and fluorescence intensity analysis of TUNEL (green) and DAPI (blue) of mammary tissues, bar = 50 μm. **C** GSEA plots of indicated gene signatures were enriched in antigen processing and presentation. **D** Heat map of differentially

expressed genes in the antigen presentation pathway. **E** Cytokine protein levels (INF-γ, IL-1β, IL-6, IL-10 and TNF-α) in mammary gland by ELISA. **F** Dendritic cells ($CD11b^+CD11c^+MHCII^+CD86^+$), CD4 T cells ($CD3^+CD4^+$), and CD8 T cells ($CD3^+CD8^+$) present in inguinal lymph nodes. **G** RT-qPCR measurement of mRNA levels of HSP70, HSP90, CRT, and HMGB1 in the treated mammary glands 24 hours after different treatments. **H** Flow cytometry analysis of natural killer cells ($CD3^-CD49^+$), neutrophils ($CD11b^+Ly6G^+$), macrophages ($CD11b^+F4/80^+$), B lymphocytes ($CD3^-CD19^+$), CD4 T cells ($CD3^+CD4^+$), and CD8 T cells ($CD3^+CD8^+$) in the mammary microenvironment. The data were expressed as means ± SD, $^{\#}p > 0.05$, $^{*}p < 0.05$, $^{**}p < 0.01$, $^{****}p < 0.0001$.

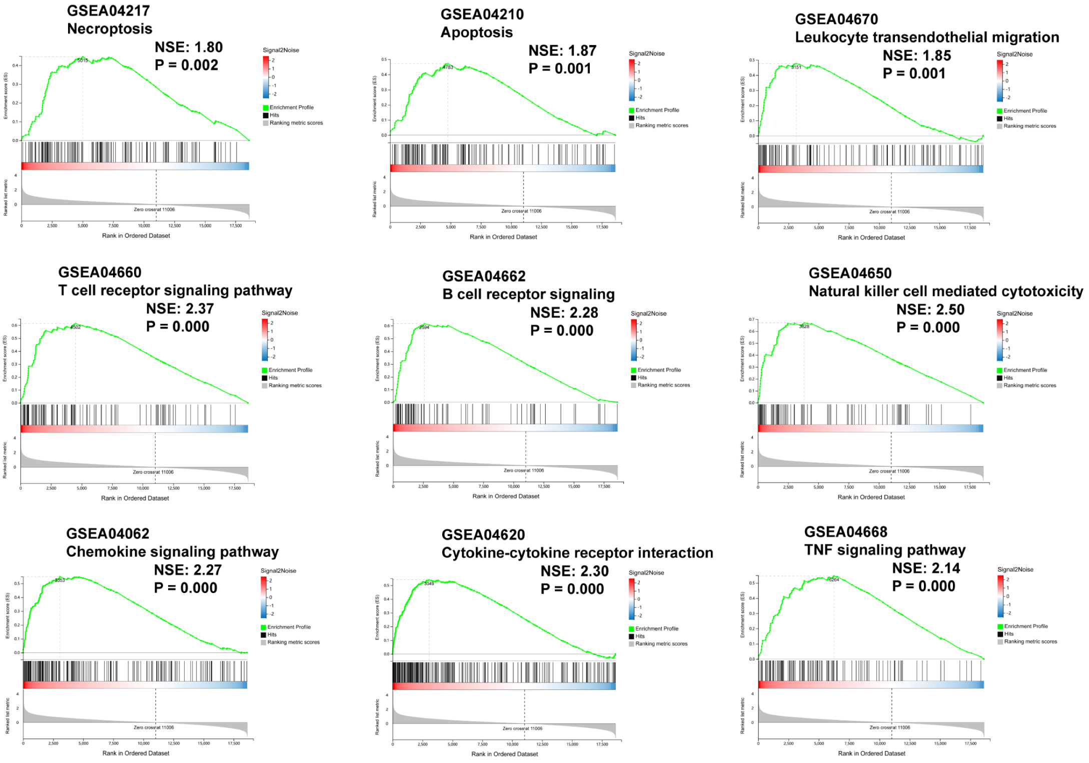

Supplementary Figure 3 GSEA analysis of signaling pathways associated with immune activation. GSEA was performed using RNA sequence data.

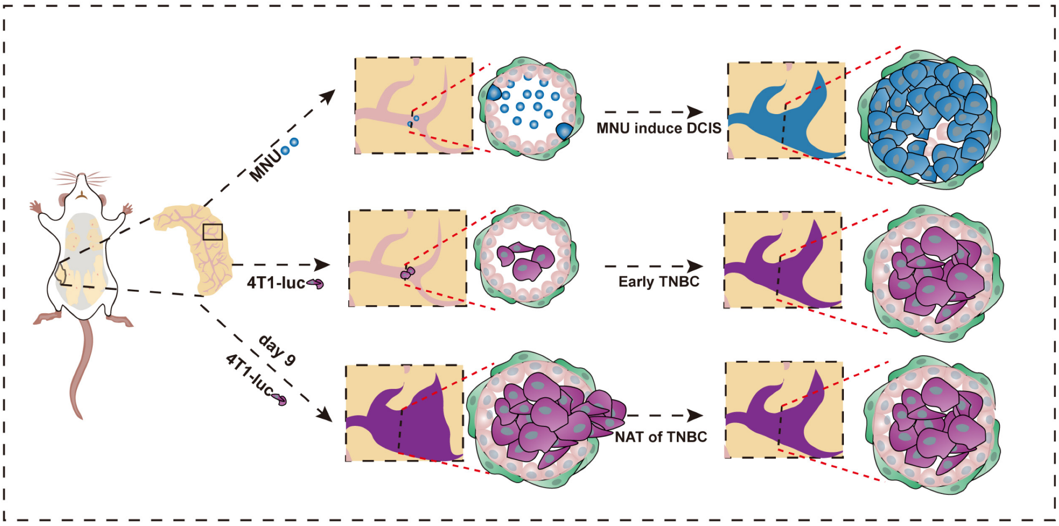

Supplementary Figure 4 Evaluation of antitumor effects of duct ablation on BC treatment and prevention in animal models. The tumor models included the MNU-induced DCIS model, early-stage TNBC model, and neoadjuvant TNBC model.

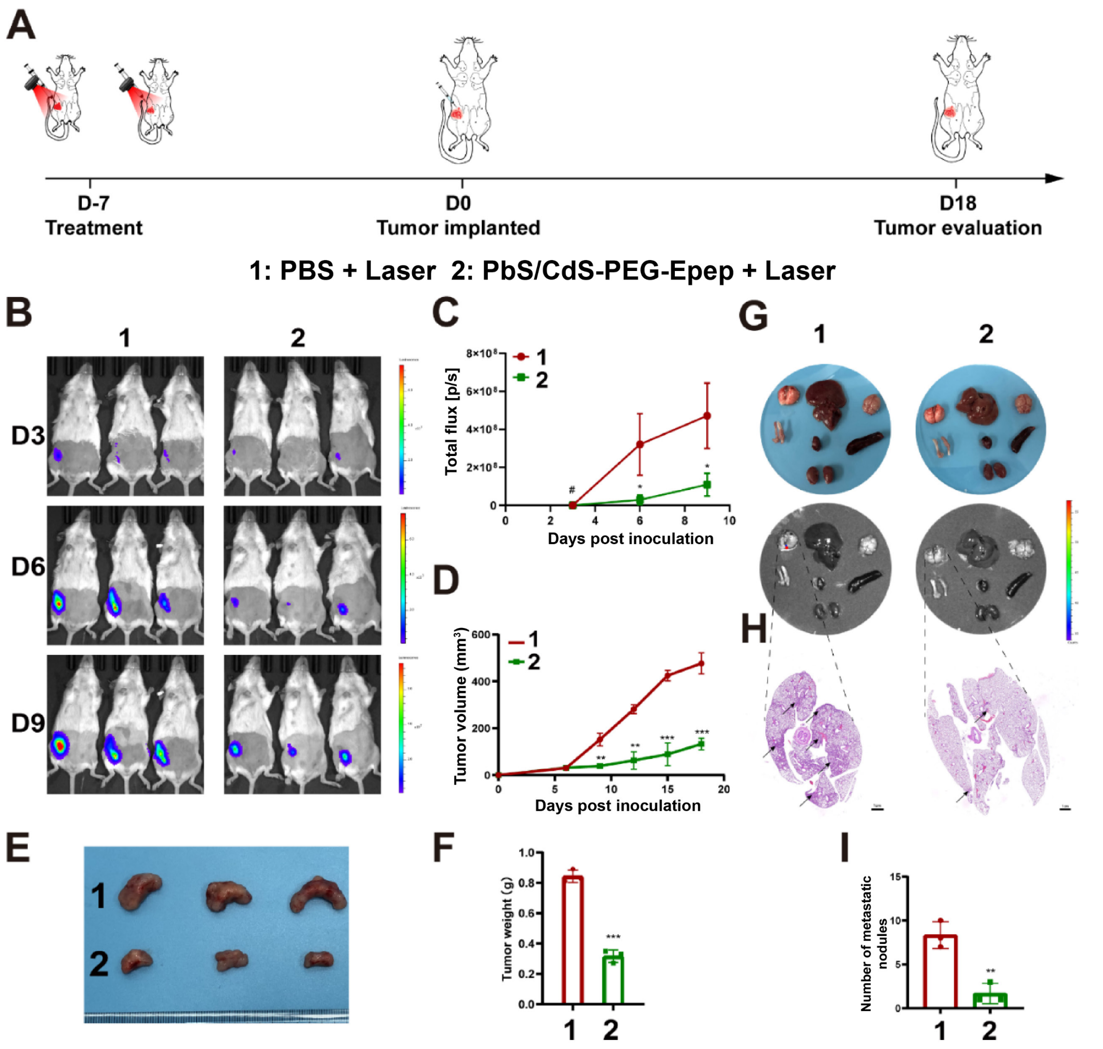

Supplementary Figure 5 Targeted mammary ablation *in situ* for BC prevention in 4T1-luc. A Schematic diagram of the animal experiments (n = 3). **B, C** *In vivo* imaging and fluorescent signal curve of PBS and PbS/CdS-PEG-Epep treatment groups on day 3, 6, and 9 after tumor inoculation. **D** The growth curve of 4T1-luc graft tumor volume. **E** Tumor tissues *in vitro* on day 18 after tumor inoculation. **F** The weight of tumors in two groups is shown. **G** Imaging of major organs under light microscopy and bioluminescence. **H, I** Micrograph of H&E stained lung section and statistical analysis of the number of metastases in the two treatment groups; black arrows indicate metastases. bar = 1 cm, data were expressed as means ± SD, $^{\#}p > 0.05$, $^{*}p < 0$ 05, $^{**}p < 0.01$, $^{***}p < 0.001$.

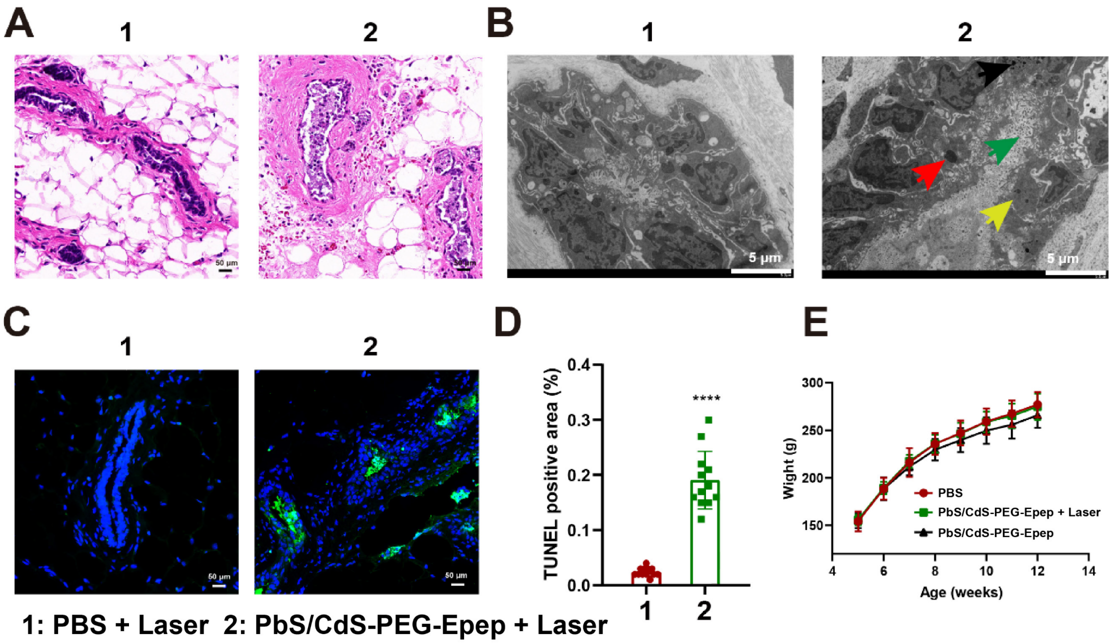

Supplementary Figure 6 Targeted mammary imaging and photothermal effects of PbS/CdS-PEG-Epep QDs in rats. A After intraductal injection of PBS and PbS/CdS-PEG-Epep QDs to rats and irradiation with 808 nm laser for 10 mins, rat mammary tissue sections were stained with H&E, bar = 50 μm. **B** TEM of rat mammary ducts after photothermal damage; the green arrow indicates lost cilia, the black arrow indicates autophagolysosomes, the yellow arrow indicates apoptotic bodies, the red arrow indicates chromosomal edge sets and pachysis; bar = 5 μm. **C, D** Immunofluorescence and fluorescence intensity analysis of TUNEL (green) and DAPI (blue) of mammary tissues, bar = 50 μm, the date was expressed as mean ± SD, $^{****}p < 0.0001$. **E** The body weight curve represents the average weight change of multiple rats, the data were expressed as mean ± SD (n = 10).

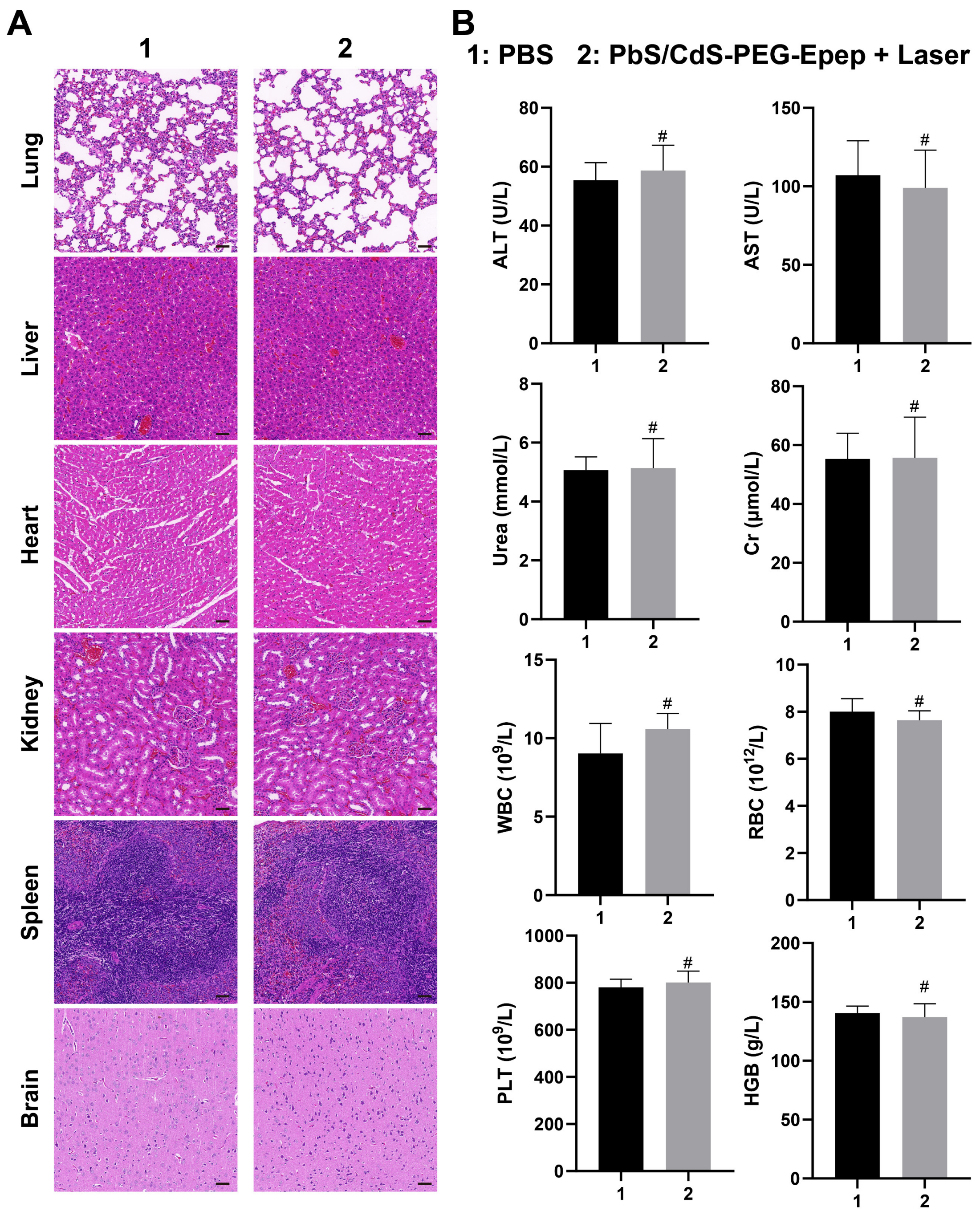

Supplementary Figure 7 Toxicity and safety assessment of PbS/CdS-PEG-Epep + Laser by i.duc administration to Rat. A H&E staining images of the lung, liver, heart, kidney, spleen, and brain after i.duc injection of PBS and PbS/CdS-PEG-Epep + Laser for 52 weeks (n = 5), bar = 50μm. **B** alanine aminotransferase (ALT), aspartate aminotransferase (AST), Urea, creatinine (Cr), white blood cell (WBC), red blood cell (RBC), platelets (PLT), hemoglobin (HGB). The data were expressed as means ± SD. $^{\#}p > 0.05$.
